# Environmental Control of Single‐Molecule Junction Evolution and Conductance: A Case Study of Expanded Pyridinium Wiring

**DOI:** 10.1002/anie.202013882

**Published:** 2021-01-07

**Authors:** Štěpánka Nováková Lachmanová, Viliam Kolivoška, Jakub Šebera, Jindřich Gasior, Gábor Mészáros, Grégory Dupeyre, Philippe P. Lainé, Magdaléna Hromadová

**Affiliations:** ^1^ Department of Electrochemistry at Nanoscale J. Heyrovský Institute of Physical Chemistry of the Czech Academy of Sciences Dolejškova 3 182 23 Prague 8 Czech Republic; ^2^ Research Centre for Natural Sciences Hungarian Academy of Sciences Magyar tudósok krt. 2 1117 Budapest Hungary; ^3^ Université de Paris ITODYS, CNRS 75006 Paris France

**Keywords:** expanded pyridiniums, molecular electronics, scanning tunneling microscopy, single-molecule conductance, solvent gating

## Abstract

Environmental control of single‐molecule junction evolution and conductance was demonstrated for expanded pyridinium molecules by scanning tunneling microscopy break junction method and interpreted by quantum transport calculations including solvent molecules explicitly. Fully extended and highly conducting molecular junctions prevail in water environment as opposed to short and less conducting junctions formed in non‐solvating mesitylene. A theoretical approach correctly models single‐molecule conductance values considering the experimental junction length. Most pronounced difference in the molecular junction formation and conductance was identified for a molecule with the highest stabilization energy on the gold substrate confirming the importance of molecule–electrode interactions. Presented concept of tuning conductance through molecule–electrode interactions in the solvent‐driven junctions can be used in the development of new molecular electronic devices.

## Graphical abstract

Single‐molecule junction conductance can be efficiently tuned by solvent environment. A series of expanded pyridinium molecular wires gave higher conductance with fully extended junction geometries in solvating water environment as opposed to mesitylene solvent. Environmental control efficiently operates the molecule–electrode interactions.

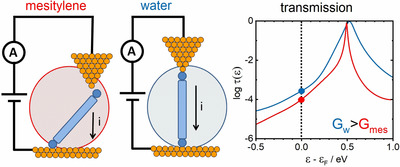

## Introduction

For living systems, it is a common place to state that solvent (water) fully contributes to supramolecular assembling processes. An open issue is the extent to which this assertion remains relevant for man‐made systems including cross‐scale hybrid assemblies identified as molecular junction (MJ) nanodevices. When these functional assemblies are operating in a solvent‐based environment at room temperature (contrary to ultra‐high vacuum and low temperatures), such conditions are likely to impact on the active molecule functioning as the charge transporting molecular wire as well as on the energetics of contact electrodes (for example Fermi energy) and molecule–electrode interactions. This is precisely the type of assessment that we report here.

An environmental control of the charge transport in single‐molecule junctions has been investigated in several recent experimental works. In all cases the emphasis was given on the explanation of the effect of solvent[^1^, ^2^, ^3^, ^4^, ^5^, ^6^, ^7^, ^8^, ^9^, ^10^, ^11^, ^12^, ^13^, ^14^, ^15^] or electrolyte (conducting salt in the solvent)[^16^, ^17^, ^18^, ^19^, ^20^, ^21^, ^22^, ^23^, ^24^, ^25^, ^26^] on the conductance value of the single‐molecule junction. In this contribution we will show that a choice of the solvent is extremely important for the junction evolution process itself, which in turn dictates the conductance value(s) obtained experimentally by the break junction methods. Chemical structure of the investigated molecule and its tendency to adsorb on the gold substrate are important factors as well.

**Figure 1:**
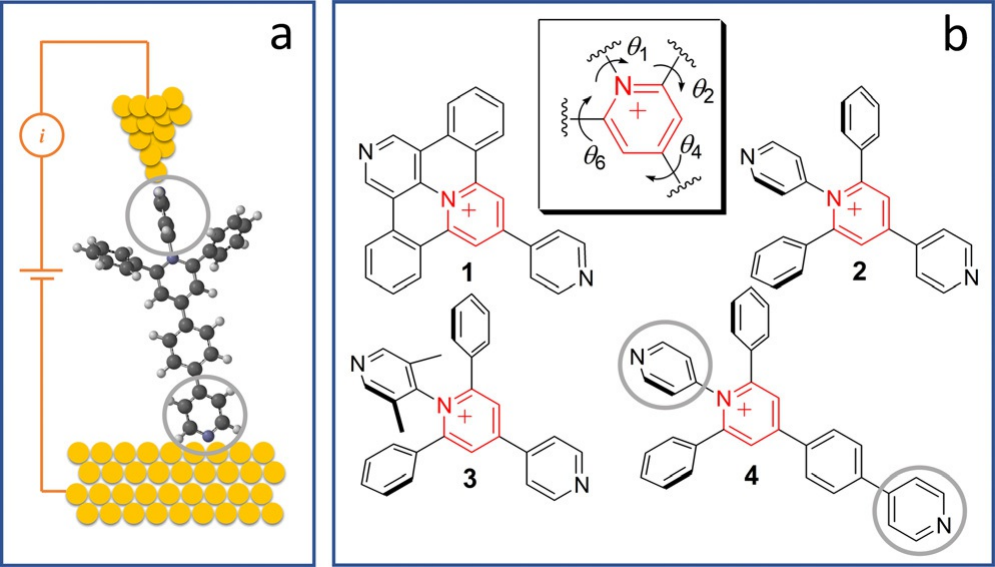
a) Representation of a single‐molecule junction with pyridine anchoring groups shown in grey circles. b) Chemical structures of expanded pyridinium molecules **1** to **4** having different degrees of conformational freedom around pyridinium core (*θ*_1_, *θ*_2_, *θ*_4_ and *θ*_6_). Counterions (BF_4_^−^) are omitted for clarity.

Break junction experiments were performed in the past in the solvent environment mainly to avoid contamination and to promote the molecular junction (Figure 1 a) formation.[^27^, ^28^] Later a suitable solvent was used to realize electrochemical gating between several single‐molecule conductance states in ON/OFF switches for molecular electronics.[^20^, ^29^, ^30^] The environmental control was also essential for the achievement of high rectification ratio in single‐molecule diodes^[4]^ and the notion of solvent gating was introduced.^[15]^

Previous reports on the solvent effect consider several reasons for the change of the conductance values including a shift of the Fermi energy due to the interaction of the solvent with the electrodes[^2^, ^5^, ^12^] or a shift in the position of the transporting orbital owing to the solvent–molecule interactions.[^6^, ^15^, ^31^]

The first systematic study of the solvent effect was given by Fatemi et al.^[2]^ who explained an increase of the single‐molecule conductance in thirteen different solvents by a shift of the work function of gold in contact with the solvent thus reducing the gap between the electrode Fermi energy and the energy of the charge transporting orbital. In their work the size of an investigated molecule was comparable with that of the solvents used. Authors claimed that direct intermolecular electrostatic interactions between the solvent and molecule did not play a role, whereas more important was the electric dipole induced in the solvent upon its adsorption to the gold. Trends in the conductance changes did not correlate either with permanent dipole moments of the solvents or with their bulk dielectric constants. Solvent dependent changes of the conductance and attenuation factor *β* in a series of oligothiophene‐based^[1]^ and oligoyne‐based^[6]^ molecular wires were also explained by a mutual shift of the electrode Fermi energy and the transporting orbital energies, though the explanation for this shift was different from that of Fatemi et al.^[2]^

Contrary to the previous work, Milan et al.^[6]^ showed that for oligoyne‐based molecular wires solvent–molecule interactions (solvation) alone can explain observed solvent effects. Bâldea^[5]^ later suggested that the solvation energy, image charges and work function changes should be considered together to quantify the solvent effect on the molecular transport in MJ nanodevices. In the electrochemically gated systems the reorganization energy of the solvent is an important factor as well.^[32]^ In such a system, Li et al.^[8]^ observed temperature dependent electron transport through single redox molecules in the aqueous electrolyte suggesting a strong coupling of the redox states to water molecules. The latest report of Tang et al.^[15]^ stressed again the importance of the solvent–molecule interactions and solvent polarity.

Herein, we selected a series of expanded pyridinium‐based molecules (pyridinium salts allowing the experiments in solvents of different polarity) with different degree of conformational freedom between the central pyridinium cation and the adjacent pyridine anchoring group (see Figure 1 b) ranging from a planar system in molecule **1** to a conformationally‐locked one in molecule **3**.^[33]^ Molecules **2** and **4** have the same pattern around the pyridinium core and differ only in their molecular length. Thus molecule **4** serves mainly as a reference compound. Synthesis and chemical characterization of these molecules are reported elsewhere.^[34]^ It is worth noting that, owing to their appealing electrophoric and structural features (namely an easily accessible LUMO, semi‐rigidity and rod‐like shape), oligomers of expanded pyridiniums have already been the subject of electrochemical investigation as model molecular wires in the context of molecular electronics.[^35^, ^36^]

The single‐molecule junction evolution and conductance G were studied in selected solvents by scanning tunneling microscopy break junction (STMBJ) method that enables repeated formation and breaking of the metal–molecule contact in the molecular junction (MJ) schematically represented in Figure 1 a. Retraction (current–distance) traces were converted to logarithmic conductance‐distance curves and corresponding 1D and 2D conductance histograms were constructed according to procedures specified elsewhere.^[37]^ Thereby, relying on this STMBJ approach, we show that beyond known effects of surrounding solvent over both the active molecular component and the contact electrodes (apexes) of MJ, this environment also sizably impacts on the conductance of MJs via their configuration, that is, by affecting molecule–electrode interfaces.

## Results and Discussion

**Figure 2:**
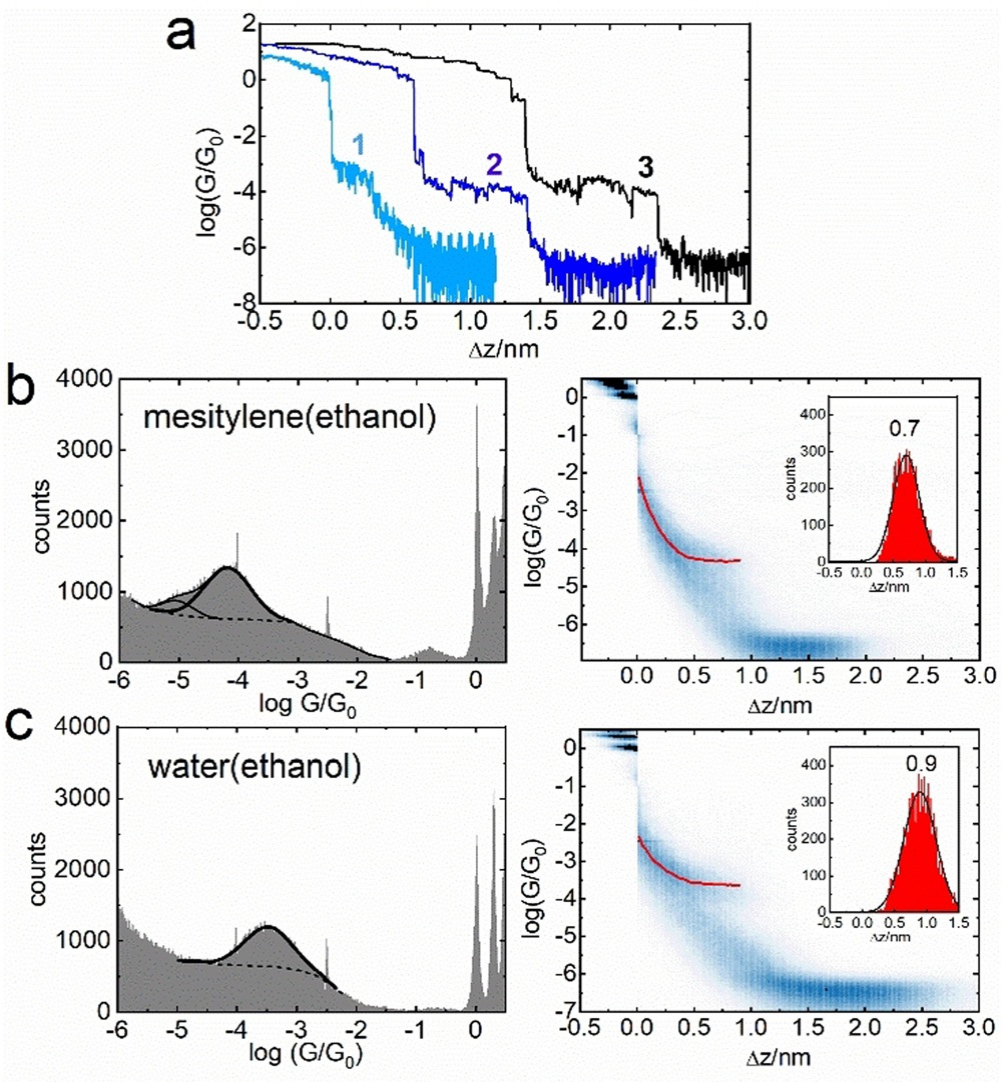
a) Representative logarithmic conductance–distance retraction curves for molecule **1** in mesitylene (1, light blue), 15 % v/v ethanol in mesitylene (2, dark blue), and 5 % v/v ethanol in water (3, black) shifted for clarity on the Δ*z* axis. b) 1D logarithmic conductance (left) and 2D logarithmic conductance–distance (right) histograms in mesitylene(ethanol) solvent. c) 1D logarithmic conductance (left) and 2D logarithmic conductance–distance (right) histograms in water(ethanol) solvent. Characteristic plateau length histograms are shown in the insets.

Figure 2 a shows representative conductance–distance (log(*G*/*G*_0_)−Δ*z*) retraction curves for molecule **1** in three different solvents. Namely, pure mesitylene, in which this molecule is only sparingly soluble (curve 1), and for 0.2 mM solution of **1** in mesitylene‐based (curve 2) and water‐based (curve 3) solvent mixtures with some added ethanol, which was the solvent of choice for preparation of the stock solution. These solvent mixtures will be hereafter referred to as mesitylene(ethanol) and water(ethanol). Based on these representative curves there is a clear indication that the plateau length for each MJ is different.

Logarithmic conductance–distance curves show plateaus at integer multiples of quantum conductance *G*_0_=77.5 μS followed by either purely tunneling current (no molecule bridging the junction) or by additional plateau(s) corresponding to the MJ conductance (see Figure 2 a). Measurements in the solvents (in the absence of investigated molecules) show purely tunneling currents and were used to provide a snap‐back distance value, which needs to be added to the characteristic plateau length Δ*z* to get the experimental MJ length *z*_exp_. Further experimental details, statistical analysis of the STMBJ data for solvents and molecules **1** to **4** are given in Sections 1 to 4 of the Supporting Information.

Figure 2 b shows statistically significant 1D logarithmic conductance (left graph) and 2D logarithmic conductance‐distance (right graph) histograms for molecule **1** in mesitylene(ethanol) solvent. Figure 2 c shows these 1D and 2D histograms for **1** in water(ethanol) environment. The insets in the right graphs show the characteristic plateau length Δ*z* histograms for each solvent mixture used. Thus, the analysis of a large ensemble of the conductance–distance curves confirms that the plateau length is shorter in mesitylene(ethanol) compared to water(ethanol) solvent, whereas the conductance of the MJ of molecule **1** in water(ethanol) environment is higher than in mesitylene(ethanol). The effect of solvent on the MJ evolution was studied on the entire series of selected molecules **1** to **4** and Table 1 gathers experimentally obtained single‐molecule conductance values on the logarithmic scale log(*G*/*G*_0_)^exp^, experimental MJ length values *z*_exp_ and junction formation probabilities in mesitylene(ethanol) and water(ethanol) solvents obtained as an average of several data sets of retraction traces (each between 2000–4000 traces). The representative 1D logarithmic conductance, 2D logarithmic conductance‐distance and characteristic plateau length histograms are shown for all molecules in Sections 3 and 4 of the Supporting Information. In some cases, two conductance states have been found (see Figure 2 b left), whereas the analysis of more prominent one was used in the following discussion. Data related to charge transport in MJ of molecule **1** in pure mesitylene have been reported elsewhere.^[11]^

**Table 1:** Experimental and theoretical single‐molecule conductance values expressed as log(*G*/*G*_0_)^exp^ and log(*G*/*G*_0_)^th^, experimental *z*_exp_ and theoretical *z*_th_ molecular junction length and junction formation probability JP for molecules **1** to **4** in different solvent mixtures.^[a]^

MJ	log(*G*/*G*_0_)^exp^	log(*G*/*G*_0_)^th^	*z*_exp_ [nm]^[b]^	z_th_ [nm]^[c]^	JP [%]^[d]^	log(*G*/*G*_0_)^exp^	log(*G*/*G*_0_)^th^	*z*_exp_ [nm]^[b]^	z_th_ [nm]^[e]^	JP [%]^[d]^
	mesitylene (ethanol)					water (ethanol)				
1	−4.1±0.5	−4.0	1.1±0.2	1.1	42	−3.5±0.5	−3.6	1.3±0.3	1.3	62
2	−4.7±0.5	−4.8	1.0±0.2	1.0	34	−4.5±0.4	−4.6	1.3±0.3	1.3	76
3	−4.7±0.5	−4.6	1.1±0.2	1.0	33	−4.6±0.5	−4.4	1.3±0.3	1.3	68
4	−5.2±0.5	−5.2	1.2±0.3	1.2	48	−5.3±0.3	−5.2	1.5±0.3	1.7	74

[a]  Interval ± represents half of FWHM of the gaussian fit of the peak in the corresponding histogram. [b] Experimental MJ length obtained from characteristic plateau length Δ*z* histograms corrected for a snap‐back distance; *z*_exp_=Δz*+0.4 nm. [c] Theoretical MJ length *z*_th_ was fixed to the *z*_exp_ value. [d] Junction formation probability shows percentage of retraction curves with at least one (high, low) conductance plateau in the ensemble. [e] Theoretical MJ length obtained from the geometry optimized MJ configuration.

The experimental MJ length *z*_exp_ was obtained as the most probable plateau length value Δz* in the characteristic plateau length Δ*z* histogram corrected for a snap‐back distance equal to 0.4 nm (see Section 2 in the Supporting Information). Analysis of the experimental MJ length indicates that for all studied molecules, junctions break at shorter distances in the mesitylene(ethanol) as compared to the water(ethanol) environment (see Table 1).

From the junction formation probability (JP) analysis of the retraction curves (examples shown in Figure 2 a) one can conclude that JP of **1** in pure mesitylene is the lowest and amounts to only 15 % and increases to 42 % in mesitylene(ethanol) and 62 % in water(ethanol) solvents. The same trend was observed for all studied molecules **1** to **4**. On average the change of the solvent from mesitylene(ethanol) to water(ethanol) almost doubles the MJ formation probability, see Table 1. In summary, single‐molecule conductance of molecules **1** to **3** is higher in water(ethanol) compared to mesitylene(ethanol), the largest difference being for molecule **1**. On the contrary, single‐molecule conductance for **4** is slightly higher in mesitylene(ethanol) and follows a predicted pattern from tunneling theory, that is, that shorter MJ geometries should have higher conductances compared to longer ones.

As mentioned above, the effect of solvent on the single‐molecule conductance was explained either by the shift of the Fermi energy due to the interaction of the solvent with the electrodes[^2^, ^5^, ^12^] or by the changing energy of the transporting orbital with respect to Fermi energy due to the solvent‐molecule interactions.[^6^, ^15^, ^31^] These factors can be easily incorporated within the framework of the Newns–Anderson model as was done by Bâldea.[^5^, ^38^]

In the present work we used the density functional theory (DFT) combined with a non‐equilibrium Green's function (NEGF) approach to calculate single‐molecule conductance values for MJ models that include explicitly solvent molecules and experimentally measured MJ lengths. The model was developed from vacuum to that either including 6 mesitylene or 42 water molecules as the solvent surrounding the expanded pyridinium molecule without further geometry restrictions. Later, the distance between two gold electrodes was adjusted to a value that corresponded to the experimentally obtained MJ length *z*_exp_. All computational details and model development steps are given in Section 5 of the Supporting Information. Sections 6 to 10 of the Supporting Information show the optimized MJ geometries, transmission functions *τ*(*ϵ*) and molecule‐localized charge transporting orbitals and their energies for MJs of **1** to **4** in vacuum, mesitylene and water environments. For all used model systems transmission functions were computed at zero‐bias approximation and used to calculate theoretical log(*G*/*G*_0_)^th^ values employing Landauer formula *G*=*G*_0_ *τ*(*ϵ*_F_), where *τ*(*ϵ*_F_) is the transmission function at the Fermi energy *ϵ*_F_ of the gold electrodes.[^39^, ^40^] It is known experimentally that the position of Fermi energy *ϵ*_F_ depends on the environment (*ϵ*_F_=−5.1 eV in vacuum). The *ϵ*_F_ of gold in contact with water is shifted by 0.6±0.1 eV[^5^, ^41^] and therefore the value of *ϵ*_F_=−4.5 eV was used for water in this work.

The *ϵ*_F_ of gold in contact with mesitylene is not experimentally known and thus we decided to use the value that gives the closest agreement between theoretical and experimental G values. The same approach was used previously by Milan et al.^[6]^ Section 11 of the Supporting Information summarizes theoretically obtained single‐molecule conductance values for molecules **1** to **4** (Supporting Information, Table S2) and the effect of the choice of *ϵ*_F_ value on log(*G*/*G*_0_)^th^ values (Supporting Information, Tables S3 and S4).

**Figure 3:**
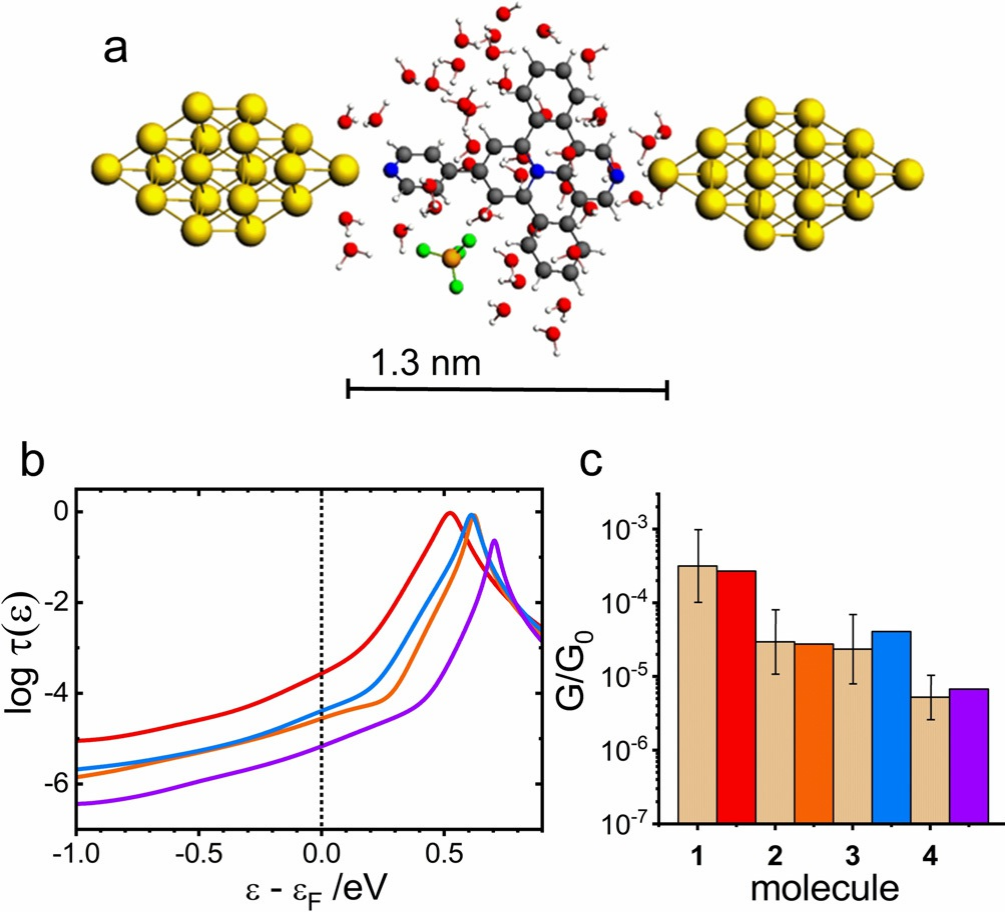
a) Geometry‐optimized MJ configuration for molecule **1** in water. b) Logarithm of transmission *τ*(*ϵ*) as a function of *ϵ*−*ϵ*_F_ for molecule **1** (red), **2** (orange), **3** (blue), and **4** (violet) for *ϵ*_F_=−4.5 eV. c) Comparison of the experimental G/G_0_ values (dotted bar with ± interval) with theoretical ones (colored bars) on the logarithmic scale.

**Figure 4:**
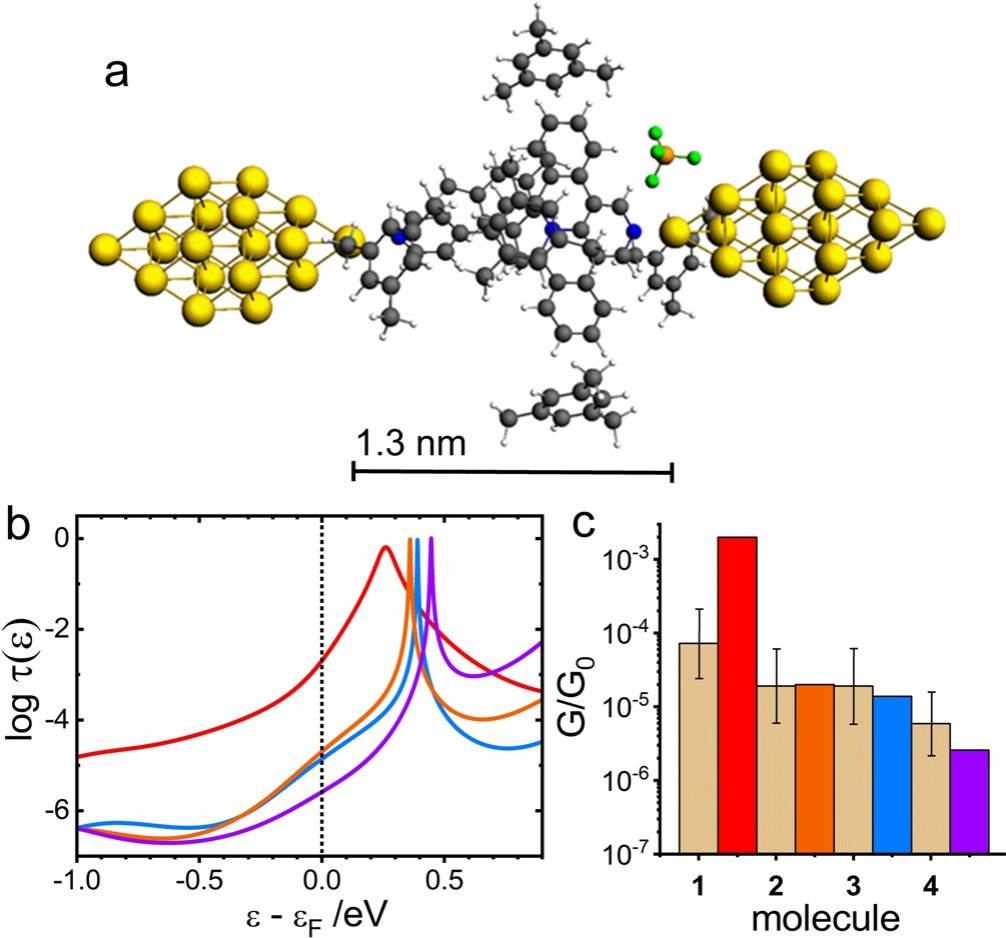
a) Geometry optimized MJ configuration for molecule **1** in mesitylene. b) Logarithm of transmission *τ*(*ϵ*) as a function of *ϵ*−*ϵ*_F_ for molecule **1** (red), **2** (orange), **3** (blue), and **4** (violet) for *ϵ*_F_=−4.7 eV. c) Comparison of the experimental *G*/*G*_0_ values (dotted bar with ± interval) with theoretical ones (colored bars) on the logarithmic scale.

**Figure 5:**
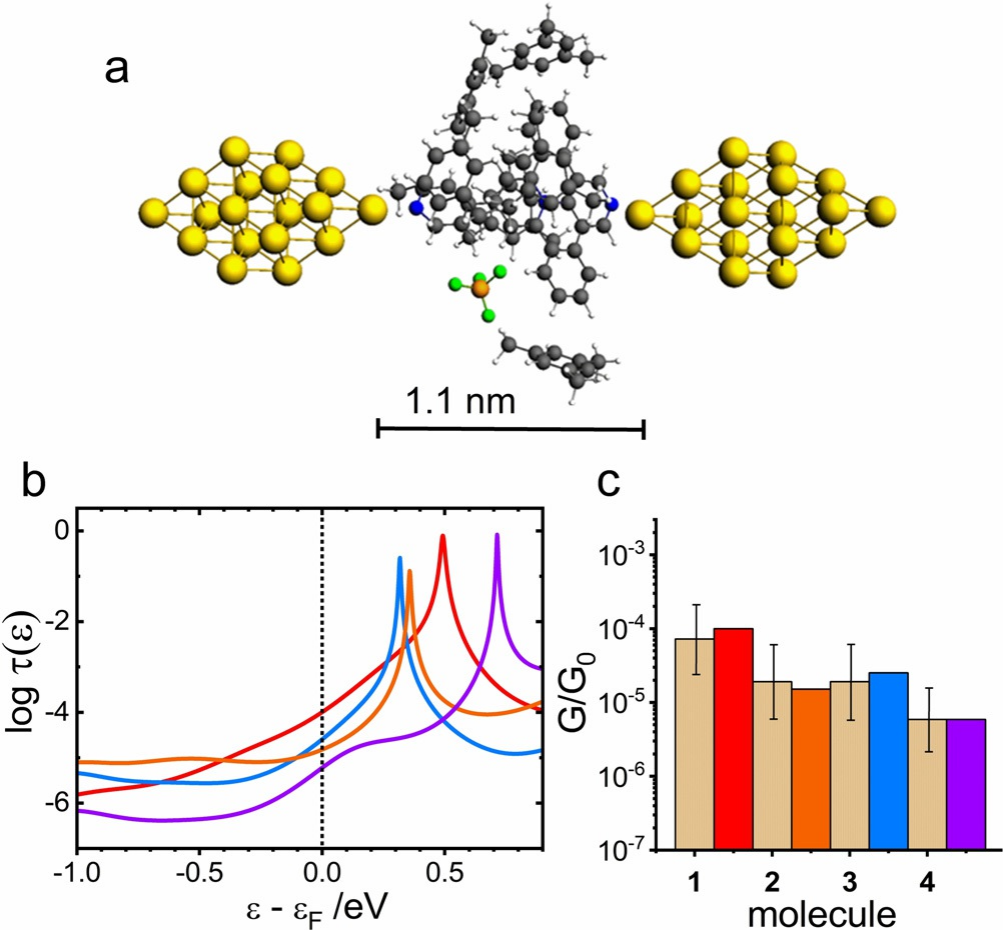
a) Geometry optimized MJ configuration for molecule **1** in mesitylene restricted to *z*_exp_ junction length. b) Logarithm of transmission *τ*(*ϵ*) as a function of *ϵ*−*ϵ*_F_ for molecule **1** (red), **2** (orange), **3** (blue), and **4** (violet) for *ϵ*_F_=−4.8 eV. c) Comparison of the experimental *G*/*G*_0_ values (dotted bar with ± interval) with theoretical ones (colored bars) on the logarithmic scale.

Theoretical calculations confirmed that in all studied systems (including both environments) LUMO is the charge transporting orbital (see transmission curves in Figures 3 to 5) consistently with previously reported calculations for molecules terminated by pyridine anchoring groups.[^42^, ^43^] Molecule localized transporting orbitals (LUMO) are shown in Sections 6 to 9 of the Supporting Information.

Before assessing the solvent effect explicitly, it is worth comparing intrinsic features of active molecules **1** to **4** regardless of their solvent environment. For molecule **1** (contrary to molecules **2** and **4**) LUMO does not remain confined along the main molecular axis that involves pyridine anchoring termini (see transporting orbitals in Sections 6 to 9 of the Supporting Information for vacuum). In the case of molecule **1** LUMO spreads a little out of the longitudinal rod‐like domain, over laterally fused phenyl rings. The pyridine anchor is embedded within the fused scaffold of **1** and is practically coplanar to pyridinium core ring (*θ*_1_=0.2° in Figure S5a of the Supporting Information, for the definition of *θ*_1_ see Figure 1 b and Figure S5a) leading to almost fully conjugated system.^[44]^ According to cos^2^ *θ* rule,[^43^, ^45^, ^46^, ^47^] the LUMO energy of **1** becomes lower compared to related species **2** and **3** containing tilted pyridine moiety (*θ*_1_=67° for **2** and 78° for **3**, see Figures S8a and S11a of the Supporting Information). This stabilization brings LUMO energy closer to the Fermi energy *ϵ*_F_ of gold electrodes (see Table S1 of the Supporting Information) for **1** and supports the observation of the highest experimental log(*G*/*G*_0_) for this molecule in both solvent mixtures (see Table 1). In summary, computational results in vacuum (see Table S2 of the Supporting Information) confirm the decrease of log(*G*/*G*_0_) going from molecule **1** to **4** in the break junction experiment.

The solvent effect was evaluated by the MJ model that explicitly incorporated solvent molecules into the molecular junction. Figure 3 shows a summary of our theoretical results obtained for single‐molecule junctions of **1** to **4** in water without any MJ length restrictions. Figure 3 a shows the MJ geometry with theoretical MJ length equal to 1.3 nm for molecule **1**. Theoretically obtained MJ length values *z*_th_ are 1.3 nm for molecules **1** to **3** and 1.7 nm for molecule **4** in a good agreement with experimental MJ length values *z*_exp_ (see Table 1) with only slightly higher value for the longest molecule **4**. Computed log(*G*/*G*_0_)^th^ values are also in very good agreement with the experiment (see Figure 3 c, Table 1 and Section 11 of the Supporting Information). Figure 3 b shows the corresponding transmission functions with LUMO being the charge transporting orbital.^[34]^ We were able to reproduce our experimental results in water(ethanol) by an explicit inclusion of the water molecules and by considering a shift of the Fermi energy of gold electrodes in contact with water to a value obtained experimentally in an independent experiment.

The identical procedure was used for transmission function calculations in mesitylene solvent. In this case only 6 solvent molecules were used to keep the complexity of the system at the same level as was the case of water. Theoretical MJ length values for geometry optimized MJs of **1** to **4** in mesitylene stayed the same as those reported for water after the geometry optimization of the entire metal–molecule–metal system (compare Figure 3 a and Figure 4 a for molecule **1**). The MJ geometries for other three molecules are shown in Sections 7 to 9 of the Supporting Information. However, the computed log(*G*/*G*_0_)^th^ values show much larger deviations from the experimental values as those for water environment, the largest difference being for molecule **1** (see Figure 4 c).

As discussed above, it is known that single‐molecule conductance values are dependent on a torsion angle between two covalently bound aromatic units.[^43^, ^45^, ^46^, ^47^] Applying this concept to molecule **1** we have performed transport calculations with systematically varied torsion angle *θ*_4_ between the planar pyridinium center and the adjacent pyridine anchoring group connected in a *para* position to the pyridinium center (for the definition of torsion angle *θ*_4_ see Figure 1 b and Section 12 of the Supporting Information). In an unconstrained system *θ*_4_ is 34.7° giving log(*G*/*G*_0_)^th^=−2.7, which is far from the experimentally observed value −4.1±0.5 (compare red bar with a dotted one in Figure 4 c). The closest agreement between log(*G*/*G*_0_)^th^ and log(*G*/*G*_0_)^exp^ was found for *θ*_4_ fixed to the unlikely value of 75°, in which case log(*G*/*G*_0_)^th^ was −4.0.

We have shown that the discrepancy between experimentally obtained and theoretically predicted charge transport characteristics for MJ of **1** in mesitylene solvent can be rationalized by changes of the torsion angle between planar pyridinium core and the pyridine anchor in the *para* position to this core. However, the theoretical MJ geometry corresponds to a fully extended MJ, which was not observed in the experiment performed in mesitylene. We have already shown (see Figure 2 and Table 1) that the experimental MJ length values are shorter in mesitylene(ethanol) compared to those in the water(ethanol) solvent. Therefore, we used these *z*_exp_ values as the constrain parameter to obtain new geometry optimized MJ configurations in the mesitylene solvent for the description of the experimental data in mesitylene(ethanol) for molecules **1** to **4**. Figure 5 a shows the MJ configuration for molecule **1** obtained for MJ length fixed to *z*_th_=*z*_exp_ obtained in mesitylene(ethanol) environment which is equal to 1.1 nm (see Table 1). The MJ configurations for all studied molecules at the experimentally observed MJ lengths are summarized in Section 13 of the Supporting Information. Figure 5 b shows the corresponding theoretical transmission functions and Figure 5 c compares the theoretical and experimental log(*G*/*G*_0_) values for MJs of molecules **1** to **4**. Theoretical conductances obtained after constraining the MJ length to the experimental *z*_exp_ value reproduce log(*G*/*G*_0_)^exp^ without the need to invoke other constraints like the torsion angle discussed above for the fully extended MJ of molecule **1**. Incidentally, the angle *θ*_4_ for molecule **1** is 28.1° in the MJ configuration shown in Figure 5 a meaning that the overall structure of molecule **1** is more planar compared to the fully extended MJ shown in Figure 4 a where the torsion angle *θ*_4_ equals to 34.7°. In spite of this planarity the single‐molecule conductance of **1** in this MJ configuration is lower (log(*G*/*G*_0_)^th^=−4.0, see Figure 5 c and Table 1) than the conductance computed for the fully extended molecular junction (log(*G*/*G*_0_)^th^=−2.7, Figure 4 c). This finding seemingly contradicts the generally accepted cos^2^ *θ* rule,[^45^, ^46^, ^47^] but can be explained by a smaller coupling strength between the molecule‐localized transporting orbital (LUMO) and *ϵ*_F_ of the electrodes in mesitylene. The peak width of the charge transporting orbital in the transmission function is related to the coupling strength between the molecule‐localized transporting orbital and *ϵ*_F_ in the Newns–Anderson model of charge transport.^[48]^ The differences in the peak widths of the transmission functions in Figures 4 b and 5 b for molecule **1** (red curves) obtained by the combined DFT and NEGF approach indicate smaller coupling strength (narrower peak) in the experimental MJ geometry.

Overall, theoretical transmission curves for MJs in water (Figure 3 b) contain much wider transmission peaks than those for MJs in mesitylene (Figure 5 b) for all studied molecules. This means that the contact geometries at the electrode‐molecule interface are indeed solvent dependent. The distance between nitrogen atom of the pyridine anchor and the closest gold atom of the electrode is smaller in a fully extended MJs (water solvent) compared to shorter geometries (mesitylene solvent). Thus, another manifestation of the solvent effect stems from the solvent‐induced modification of the interaction between the molecule and gold electrodes leading to a different MJ configuration for each solvent used. One may rephrase this statement in such a way that solvation effects (solvent–molecule interactions) alter the molecule–electrode interactions in the process of the MJ formation and breaking. The importance of van der Waals forces between pyridine anchoring groups and gold substrate for the MJ evolution mechanics was already established by Aradhya et al.^[49]^ in the absence of the solvent by simultaneous conductance and rupture force measurements. Unfortunately, we do not have the computational abilities to simulate the entire MJ breaking process along the experimentally observed retraction curves using explicitly the solvent molecules. Nevertheless, after proper consideration of the experimental MJ length we were able to explain measured MJ conductance values simply by the solvated MJ model^[6]^ that considers solvent–molecule interactions.

Even though we are not explaining the solvent effect in terms of the individual solvent–molecule, solvent–electrode, and molecule–electrode contributions, we can still assess the last contribution because we have a series of molecules **1** to **3** with different structural arrangement around the pyridinium center and practically the same length. Calculated (in vacuum) stabilization energies of their cations on the gold(111) substrate confirmed that adsorbed cation **1** has the highest stabilization energy followed by cations **2** and **3** (see Table S6 in Section 14 of the Supporting Information). The geometry optimized structures of these adsorbates on the gold surface (Supporting Information, Figure S19) show that nitrogen atoms of both pyridine anchoring groups are in close contact with the gold substrate in cation **1** (lying flat) and there is an increase of the distance between one of the pyridine anchors and the gold substrate going from cations **1** to **3**. Thus, the role of the molecule–electrode interactions in the MJ evolution and conductance values should be most pronounced for molecule **1** as was indeed experimentally observed (see Figure 2 a).

Finally, the question as to whether aggregation^[50]^ may occur during MJ formation is worth to be addressed. In the case of branched expanded pyridiniums (**2**, **3** and **4**), the steric hindrance around pyridinium cores is likely to warrant the separation of wires beyond repelling of their cationic charge and solvation shell in the case of water solvent. In the most sensitive case of **4**, single‐crystal X‐ray crystallography of a close analogue (molecule **B^PP^** in Fortage et al.^[51]^) tells us that no noticeable π–π stacking is observed when looking at crystal packing as a limiting case for solid‐state organization, thereby indicating that branched species have no propensity to aggregation. For what concerns the fused polycyclic species **1**, crystallography of a close analogue (molecule **1^H^F** in Fortage et al.^[52]^) reveals propensity of cationic scaffolds to form stacks, which indicates that aggregation cannot be ruled out in this instance, even if rather diluted solutions (0.2 mM) were used. Noteworthy, in more unfavorable conditions, when there is no pronounced solvation of **1** by solvent molecules that is, in mesitylene environment, aggregate formation is likely to explain the second minor conductance peak that was not analyzed (see Figure 2 b). This latter may be due to two stacked molecules facing each other and since the interaction is of van der Waals type, the conductance is lower.

## Conclusion

We have observed experimentally that all studied molecules **1** to **4** form MJs with higher MJ formation probability in water(ethanol) medium than in mesitylene(ethanol) environment. The experimental MJ length corresponds to a fully extended geometry in water(ethanol) and is shorter in mesitylene(ethanol) solvent. We were able to explain all experimentally observed MJ conductance values using explicitly mesitylene and water molecules and considering different MJ geometries in these two solvents. Our theoretical results (transmission functions, MJ geometries and molecule–electrode stabilization energies) support the description of the solvent effect in which the molecule–electrode interactions must be taken into consideration in addition to the solvent–molecule and solvent‐electrode interactions considered previously. In view of a recent claim that high conductance transport pathway can be induced in MJs by applying potential to one of the electrodes to promote the molecular adsorption in a flat orientation,[^53^, ^54^, ^55^] our work further substantiates the importance of molecule–electrode interactions in the break junction measurements of the MJ conductance. Above all, it evidences the critical role of surrounding medium on the MJ formation. In particular, it shows that water favorably impacts on charge transport characteristics of cationic, redox‐active and functionally LUMO‐driven electrophilic molecular wires based on expanded pyridiniums.^[56]^

## Conflict of interest

The authors declare no conflict of interest.

## Supporting information

As a service to our authors and readers, this journal provides supporting information supplied by the authors. Such materials are peer reviewed and may be re‐organized for online delivery, but are not copy‐edited or typeset. Technical support issues arising from supporting information (other than missing files) should be addressed to the authors.

Supplementary
