# Supplementary material for: Environmental Control of Single‐Molecule Junction Evolution and Conductance: A Case Study of Expanded Pyridinium Wiring
Source: Angew Chem Int Ed Engl. 2021 Jan 7;60(9):4732–9. doi: 10.1002/anie.202013882 (PMC7986070; doi:10.1002/anie.202013882)
Supplement: Supplementary file 1 — Supplementary [file ANIE-60-4732-s001.pdf]

Angewandte Chemie
Eine Zeitschrift der Gesellschaft Deutscher Chemiker

Supporting Information

Environmental Control of Single-Molecule Junction Evolution and Conductance: A Case Study of Expanded Pyridinium Wiring

Štěpánka Nováková Lachmanová[+], Viliam Kolivoška[+], Jakub Šebera[+], Jindřich Gasior, Gábor Mészáros, Grégory Dupeyre, Philippe P. Lainé, and Magdaléna Hromadová**

anie_202013882_sm_miscellaneous_information.pdf

SUPPORTING INFORMATION

Table of Contents

1. Materials and experimental details S2
2. Statistical analysis of the STMBJ data for solvents S4
3. Statistical analysis of the STMBJ data for molecules **1** to **4** in mesitylene(ethanol) S5
4. Statistical analysis of the STMBJ data for molecules **1** to **4** in water(ethanol) S6
5. Computational details and model development S7
6. Single molecule junction of **1** in vacuum, water and mesitylene solvent S10
    6.1. Optimized molecular junction geometries S10
    6.2. Transmission functions S11
    6.3. Transporting orbitals S12
7. Single molecule junction of **2** in vacuum, water and mesitylene solvent S13
    7.1. Optimized molecular junction geometries S13
    7.2. Transmission functions S14
    7.3. Transporting orbitals S15
8. Single molecule junction of **3** in vacuum, water and mesitylene solvent S16
    8.1. Optimized molecular junction geometries S16
    8.2. Transmission functions S17
    8.3. Transporting orbitals S18
9. Single molecule junction of **4** in vacuum, water and mesitylene solvent S19
    9.1. Optimized molecular junction geometries S19
    9.2. Transmission functions S20
    9.3. Transporting orbitals S21
10. Transporting orbital energies for optimized geometries S22
11. Theoretical single molecule conductance values S23
12. Torsion angle effect S24
13. Molecular junction geometries in mesitylene at experimental junction length S25
14. Stabilization and interaction energies for adsorbed molecules S26
15. References S27

1. Materials and experimental details.

Materials. Chemical synthesis and characterization of molecules **1** to **4** has been described elsewhere.[1] Ultrapure deionized water was obtained using a Milli-Q Integral 5 water purification system (minimum resistivity 18.2 MΩ·cm and TOC of 3 ppb; Merck Millipore, France). Absolute ethanol (99.8%, molecular biology grade, AppliChem GmbH, Darmstadt, Germany and p.a. Penta, Czech Republic), 1,3,5-trimethylbenzene (mesitylene, 98% Sigma Aldrich) and nitric acid (65% p.a. Lach-Ner, Czech Republic) were used as received. Argon and nitrogen gases were obtained from Messer (99.998 % purity).

Experimental Details. Scanning tunneling microscopy probes were prepared by electrochemical etching of gold wires (0.25 mm in diameter, 99.99+ % purity Goodfellow). A polyethylene coating was used as an insulation for experiments in the polar aqueous solvent. Gold substrate (99.95 % purity, Goodfellow) was annealed by butane flame and cooled to room temperature under the stream of nitrogen to avoid the impurities. All glassware, Kalrez O−rings, PTFE liquid cell parts were cleaned in boiling 30% nitric acid, followed by repeated boiling in ultrapure water and drying at 105°C. Molecules **1** to **4** were first dissolved in pure ethanol and this stock solution was used for preparation of 0.2 mM solution of **1** to **4** in two solvents: 15% v/v ethanol in mesitylene and 5% v/v ethanol in water, respectively.

Experimental Method. The in-house modified Agilent 5500 Scanning Probe Microscope (Agilent Technologies, USA) was used for scanning tunneling microscopy break junction (STMBJ) measurements. Detailed description of the setup modification is given elsewhere.[1] This setup allows generating ramp voltage for controlling z−axis piezo position, including trigger event for detecting the metallic contact formation during approach, and another trigger event for detecting the complete breaking of the junction during probe withdrawal. The approaching and retracting rates can be varied separately. Current measurements were performed by a bipotentiostat with two bipolar tunable logarithmic I−V converters operated by a custom−designed microcontroller. Bipotentiostat controlled the potential difference (bias voltage) between two gold electrodes (the substrate and the probe) and sensed the electric current flowing through the junction. An auxiliary output channel of the bipotentiostat controlled the voltage of the piezo stack in the range of 0−32 V. The entire setup was placed in two Faraday boxes, one for the mechanical unit and the other for the controller unit in order to avoid electronic cross−talk between different functional parts. Further

details on the setup are reported elsewhere.[2−4] STMBJ measurements were done at disabled z−piezo voltage feedback control and at constant bias voltage of 130 mV between the gold probe and substrate. Experiments in water(ethanol) solvent were obtained at 37 nm/s retraction rate, those in mesitylene(ethanol) at 26 nm/s. Typically 2000 to 4000 junction formation-breaking cycles were collected to obtain statistically significant amount of data in one data set. Several data sets were collected for each molecule. Conductance−distance curves were obtained from the retraction curves using the actual calibration constants for piezo and electronic elements used employing Ohm's law for the current conversion to conductance. A combination of the in−house developed software (Delphi programming environment, RAD Studio, Embarcadero, USA) and OriginPro 9.1 (OriginLab Corporation, USA) was used for the extraction of retraction curves, their conversion to logarithmic conductance−distance $\log(G/G_0)-\Delta z$ curves and the construction of 1D and 2D histograms, where G is the conductance presented in the units of quantum conductance G_0 = 77.5 μS and Δz is the distance obtained upon aligning individual $\log(G/G_0)-\Delta z$ curves to the common point [−0.5, 0]. For 1D logarithmic conductance histograms the bin size of 0.005 was used. Bin size values of 0.05 were used for $\log(G/G_0)$ and 0.02 nm for Δz distance values for the construction of 2D logarithmic conductance−distance histograms. Typical 2D logarithmic conductance−distance histograms for two solvents used are shown in Figure S1 of the Section 2. In the absence of molecules, a characteristic decay profile is obtained referring to tunneling through the solvent. By extrapolation of the conductance profile obtained in the tunneling region to $\log(G/G_0) = 0$ (green line in Figure S1) one finds a so-called snap-back distance z_{corr} that is needed to obtain the actual length $z_{exp} = \Delta z^* + z_{corr}$ of any junction at any selected $\log(G/G_0)$ value, where Δz^* is the characteristic length obtained from a characteristic length histogram. An example of a characteristic length histogram constructed from 2D logarithmic conductance-distance histogram at $\log(G/G_0) = -5.5$ for two solvents used in this work is shown in Figure S2 (in the absence of molecules). In the presence of a molecule this type of histogram provides an experimental plateau length histogram (right panels of Figs. S3 and S4), from which an experimental molecular junction length z_{exp} can be obtained. Full description of the characteristic plateau length analysis is given elsewhere.[4] From many experiments as those shown representatively in Figure S1, the z_{corr} value was determined to be 0.4 nm independently of the solvent and retraction rate used consistently with some previous reports.[5]

2. Statistical analysis of the STMBJ data for solvents.

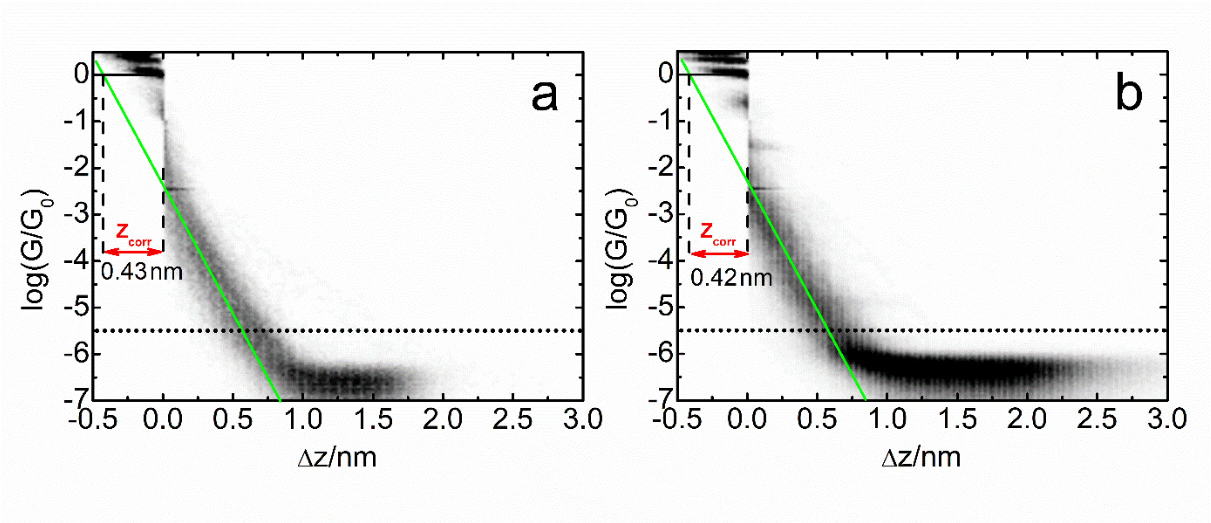

Figure S1. 2D logarithmic conductance-distance histogram for 15% v/v ethanol in mesitylene (a) and 5% v/v ethanol in water (b) solvent in the absence of investigated molecules. Retraction rate was 26 nm/s (a) and 37 nm/s (b), respectively. Green line represents linearly decreasing tunneling current on the logarithmic $\log(G/G_0)$ scale. Its extrapolation to gold atomic point contact conductance $G = G_0$ gives the snapback distance z_{corr} = 0.43 nm (a) and 0.42 nm (b), respectively.

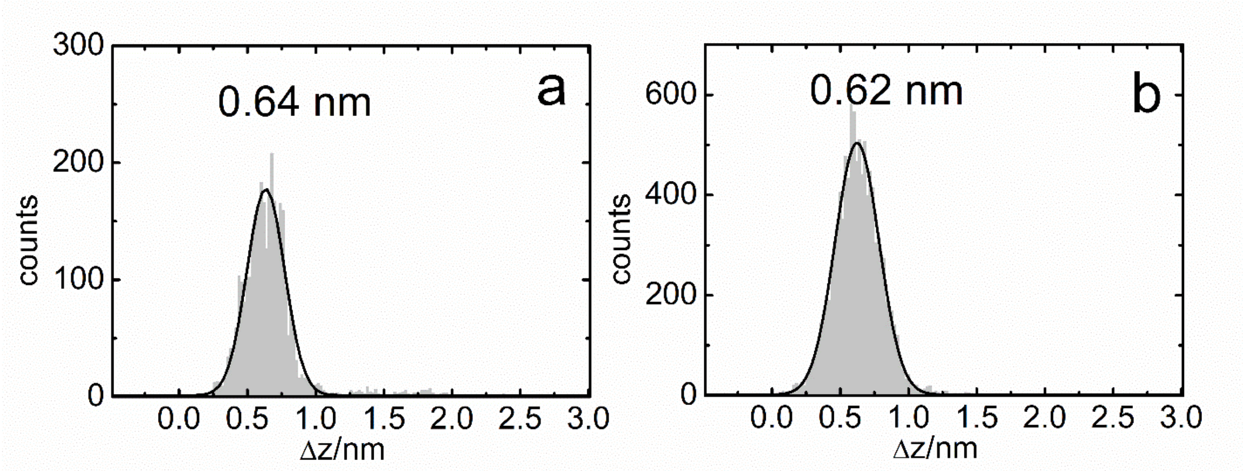

Figure S2. Characteristic length histogram obtained from data in Figure S1 as a horizontal cross-section at $\log(G/G_0) = -5.5$ represented by a dotted line in Fig. S1.

3. Statistical analysis of the STMBJ data for molecules 1 to 4 in mesitylene(ethanol)

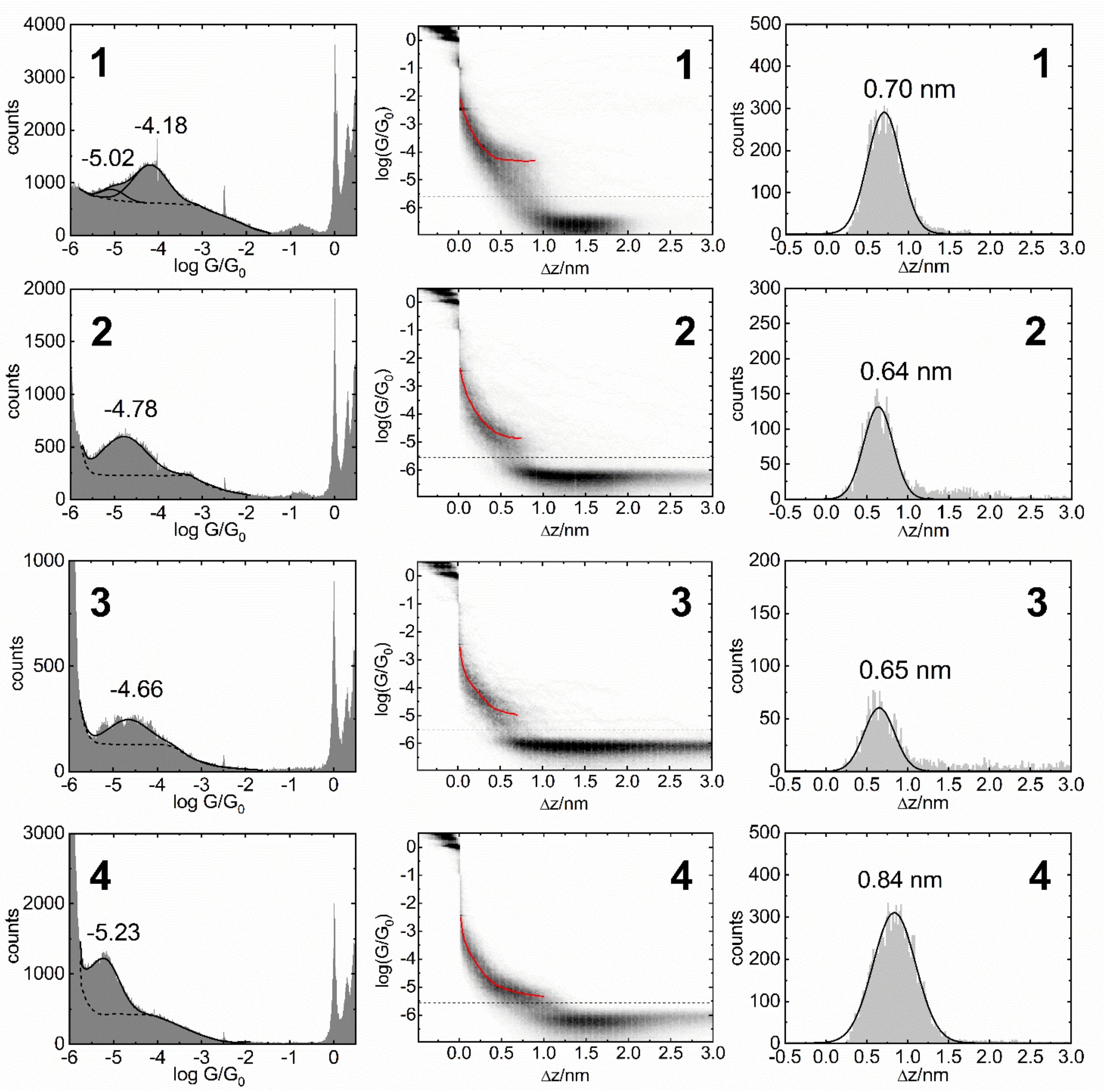

Figure S3. Representative 1D logarithmic conductance histogram (left), 2D logarithmic conductance-distance histogram (middle) and characteristic plateau length Δz histogram (right) for molecules **1** to **4**. Gold-molecule-gold junctions were formed at 25°C in 15% ethanol in mesitylene solvent containing 0.2 mM of respective compounds. Probe retraction rate was 26 nm/s. Horizontal cross-section in 2D histogram indicates the conductance values selected for Δz evaluation.

4. Statistical analysis of the STMBJ data for molecules 1 to 4 in water(ethanol)

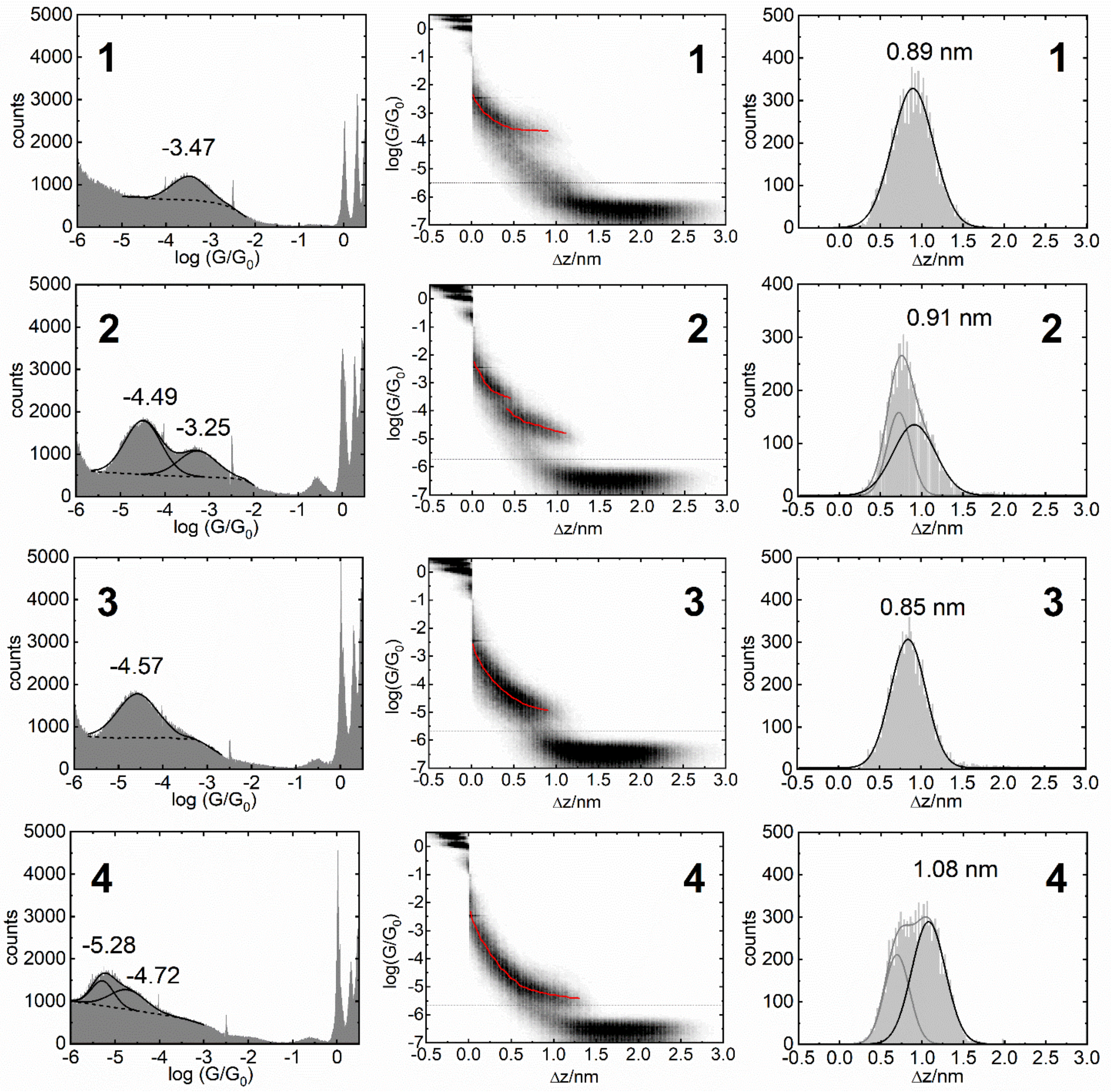

Figure S4. Representative 1D logarithmic conductance histogram (left), 2D logarithmic conductance-distance histogram (middle) and characteristic length Δz histogram (right) for molecules **1** to **4**. Gold-molecule-gold junctions were formed at 25°C in 5% ethanol in water solvent containing 0.2 mM of respective compounds. Probe retraction rate was 37 nm/s. Horizontal cross-section in 2D histogram indicates the conductance values selected for Δz evaluation.

5. Computational details and model development.

Molecular junctions (MJs) of all investigated compounds **1** to **4** were described by the computational model including two gold clusters Au_{18} (2×3×3) (representing electrodes used in the STMBJ experiment) and molecules placed between them (Figs. S5, S8, S11 and S14). The geometry optimization was performed using density functional theory (DFT) with B3LYP functional.[6,7] During the optimization the gold atoms were fixed at their experimental positions with the gold-gold bond distance of 2.885 Å.[8] A positive charge of system Au_{18}-molecule-Au_{18} was neutralized by adding BF_4^- anion into the computational model. The N, C, H, B, F atoms were described by 6-31G(d) basis set [9] and for Au atoms the double-ξ basis set LANL2DZ with quasi-relativistic effective core pseudopotential was used. [10] Geometry optimization procedure is described in detail elsewhere.[1] The equilibrium geometry of the optimized Au_{18}-molecule-Au_{18} junction in vacuum was taken as a starting point for computational modelling of all investigated systems in mesitylene and water used as the solvent environment. First solvent environment contained 6 mesitylene molecules (Figs. S5b, S8b, S11b and S14b) and the second one 42 water molecules (Figs. S5c, S8c, S11c and S14c), respectively. The number of mesitylene molecules used in our computational model was inspired by the work of Milan et al.,[11] where six mesitylene molecules were sufficient for successful interpretation of the STMBJ experimental data. The number of atoms of all water molecules surrounding the molecule in BJ computational model corresponded to the number of atoms in six mesitylene molecules included in our model (126 atoms). The geometry optimization of the solvent model was performed by ONIOM (B3LYP:PM6) methodology[12], where gold clusters, molecules **1** to **4** including BF_4^- counterions were described by B3LYP method with the same basis sets as for calculations without solvent molecules. Water and mesitylene molecules were described by a semiempirical PM6 quantum chemistry method. All geometry optimization calculations were performed using quantum chemistry package Gaussian 09.[13]

Transmission functions $\tau(\varepsilon)$[14,15] of compounds **1** to **4** (Figs. S6, S9, S12 and S15) were calculated by DFT method combined with non-equilibrium Green's function (NEGF) approach. All calculations of transmission functions were performed by using the Amsterdam Density Functional (ADF) quantum chemistry package [16] using modified B3LYP [17] hybrid exchange–correlation functional with 15% Hartree-Fock exchange and within wide-band limit (WBL) approximation.[14] The modified B3LYP functional was successfully used for interpretation of the experimentally

measured conductance of expanded pyridinium compounds.[1] Gold atoms were described by a double–ξ (DZ) basis set and for remaining atoms the double–ξ polarized (DZP) basis set was used.[18] The zero–order regular approximation (ZORA) in a scalar relativistic form as implemented in ADF program was used for description of the relativistic effects.[19]

Theoretical conductance at zero–bias approximation was calculated by using Landauer formula $G = G_0 \times \tau(\varepsilon_F)$, where G_0 is the conductance quantum and $\tau(\varepsilon_F)$ is the transmission function at the Fermi energy ε_F of the gold electrodes.[20,21] Fermi energy $\varepsilon_F = -5.1$ eV obtained experimentally for polycrystalline gold in vacuum[22] was used for preliminary calculations in vacuum (Figs. S6, S9, S12 and S15, black curves). It is known that the Fermi energy calculated by DFT method is not very reliable and the solvent affects the Fermi energy of gold contacts.[11,23] Fermi energy of gold in the water environment was experimentally measured and its value is shifted with respect to vacuum by 0.6 ± 0.1 eV.[23,24] Thus, we used a value of $\varepsilon_F = -4.5$ eV for all theoretical calculations in the aqueous environment (Figs. S6, S9, S12 and S15, red curves). Since the experimental ε_F value of gold in the presence of mesitylene is not available the value that provided the closest agreement between theoretical and experimental single molecule conductance values was selected. This approach is consistent with that of Milan *et al.*[11] For the geometry optimized fully elongated molecular junctions the value of $\varepsilon_F = -4.7$ eV was chosen for the mesitylene solvent (see Table S2 in Section 11) as it fulfils the above-mentioned requirement (Figs. S6, S9, S12 and S15, blue curves and Table S3 in Section 11). Additionally, the single molecule conductance value was calculated as a function of torsional angle ω for molecule **1** in the presence of mesitylene solvent without further constraints on the molecular junction length, see Section 12.

Molecule-localized LUMO representing the most probable charge transport channel in the single molecule junction of **1** to **4** in vacuum and with explicit inclusion of mesitylene and water solvent molecules are depicted in Figs. S7, S10, S13 and S16.

Single molecule conductance for molecules **1** to **4** in the mesitylene solvent was also calculated for geometries where the theoretical MJ distance z_{th} was set to that obtained from the experiment, see Section 11 (Table S2) and Section 13 (Figure S18). This means that the separation between the centers of apex atoms of two gold Au_{18} clusters was fixed at z_{th} + 2.5 Å since the contact distance between two gold atoms is 2.5 Å. In these short MJ geometries, the value $\varepsilon_F = -4.8$ eV provided better agreement between the experimental and theoretical single molecule conductance values, but calculations with $\varepsilon_F = -4.7$ eV give reasonable conductance values as well (see Table S4 in Section

11). At the experimental MJ length of 1.10 nm for molecule **3** we obtained $\log(G/G_0)^{th} = -3.83$ (at $\varepsilon_F = -4.8$ eV), which is far from $\log(G/G_0)^{exp} = -4.72$ observed in the experiment. Therefore, we have studied the effect of the MJ distance on the MJ conductance for this molecule to better understand such discrepancy. We found that one end of the molecule (pyridine anchor) interacts with gold cluster through π-electrons and this interaction can lead to the existence of either positive or negative quantum interference.[25] In other words, the relatively small geometrical shift of molecule **3** against the gold electrode can lead to an abrupt change in the calculated conductance. Therefore, in the main article, we postulated that the break junction geometry with $z_{th} = 1.00$ nm is more representative of the actual MJ structure for **3**. This z_{th} value is still within the experimentally obtained MJ length interval ($z_{exp} = 1.1 \pm 0.2$ nm) and leads to $\log(G/G_0)^{th}$ equal to -4.60 at $\varepsilon_F = -4.8$ eV.

The computational model for an evaluation of the interaction between molecules **1**, **2** and **3** with the gold surface consisted of two layers of gold atoms generating Au(111) surface using 64 atoms with fixed gold-gold bond distances at 2.885 Å with one molecular cation $\mathbf{1}^+$, $\mathbf{2}^+$ or $\mathbf{3}^+$ located on the upper gold layer (Figure S19). Such systems were geometry optimized using DFT functional PBE[26,27] with Grimme´s dispersion coefficient D3[28] using the resolution of identity (RI-J) approximation for Coulomb integrals.[29,30] For geometry optimization the basis set def-SVP[31] was used for N, H, C atoms and the quasirelativistic effective core pseudopotentials def-ecp[32] and the corresponding set of basis functions for Au atom. DFT calculations were performed by Gaussian 09[13] and Turbomole-7.0[30] software packages. The molecular cations alone were geometry optimized using the Turbomole program with the same procedure as mentioned above.

The stabilization ΔE (kcal/mol) and interaction ΔE_{int} (kJ/mol) energies were calculated for cations $\mathbf{1}^+$, $\mathbf{2}^+$ and $\mathbf{3}^+$ adsorbed on the gold electrode surface based on the geometry optimized structures (see Figure S19) employing PBE-D3 method (see previous paragraph). B3LYP-D3 functional[6,7,28] and 6-311G(d,p)[33] basis set for N,H,C atoms and LANL2DZ basis set based on Los Alamos effective core potential with corresponding set of basis functions for Au atom was used for more accurate results.[10] The stabilization energy ΔE was taken as the negative interaction energy with the inclusion of the basis set superposition error (BSSE) correction E_{BSSE}. The interaction energy was calculated as $\Delta E_{int} = E_{1\text{-}2} - (E_1 + E_2) + E_{BSSE}$, where $E_{1\text{-}2}$ is the energy of cation-gold complex, E_1 and E_2 are energies of the cation and gold electrode. All DFT calculations of stabilization and interaction energies were carried out in vacuum environment.

6. Single molecule junction of 1 in vacuum, water and mesitylene solvent.

6.1. Optimized molecular junction geometries.

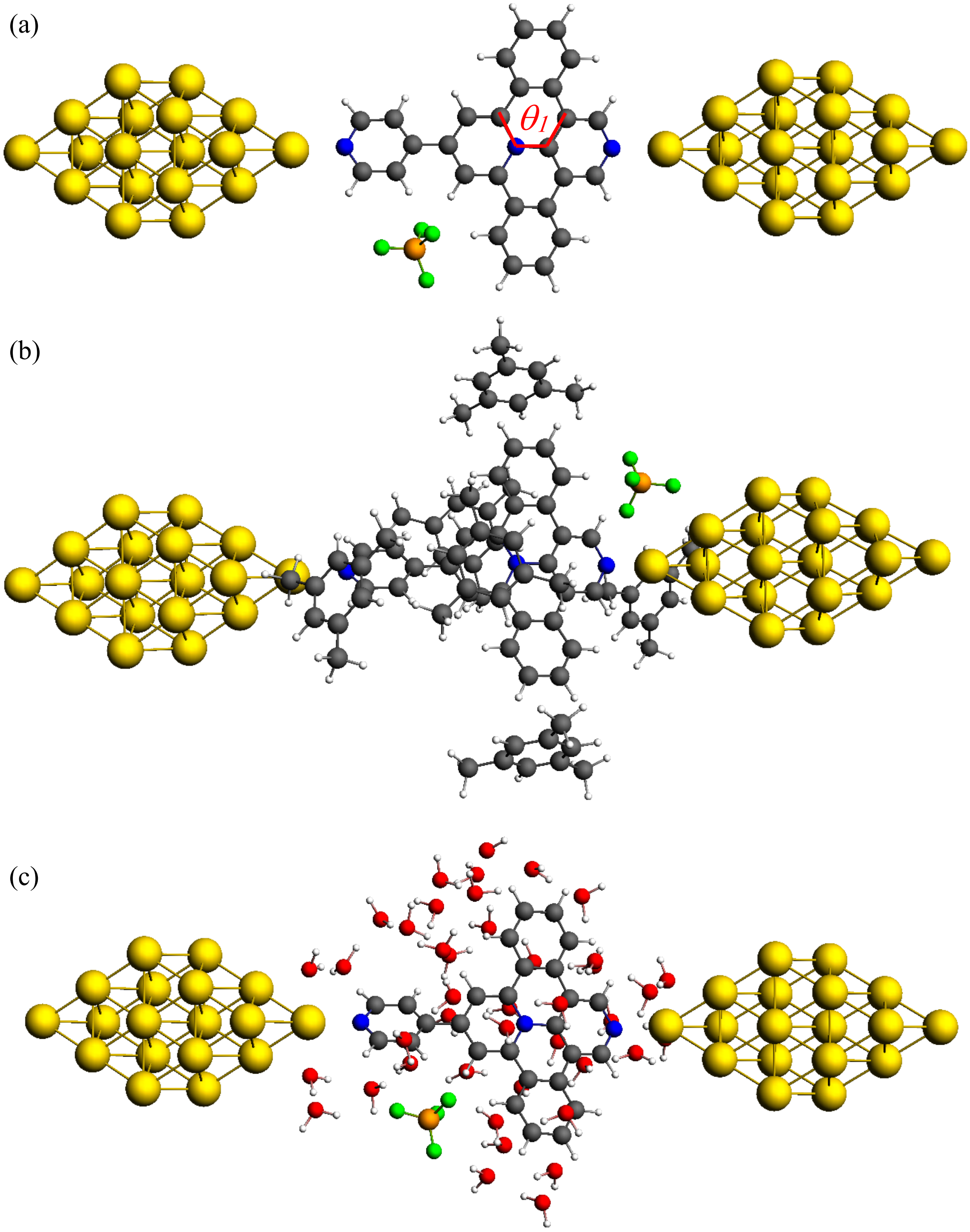

Figure S5. Molecular junction configuration for molecule **1** in vacuum (a) and with explicit inclusion of 6 mesitylene (b) and 42 water (c) molecules. The definition of torsion angle θ_1 (red).

6.2. Transmission functions

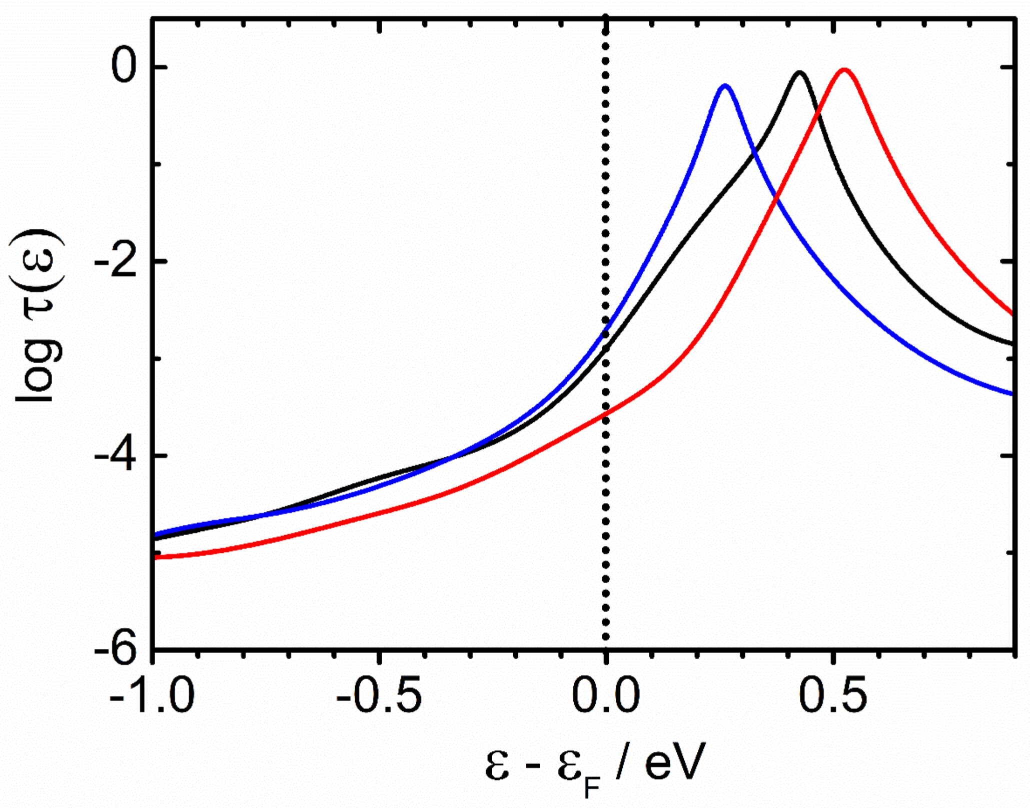

Figure S6. Transmission function for single molecule junction of **1** calculated for vacuum (black), mesitylene (blue) and water (red) environment. Energy axis was scaled using Fermi energy ε_F value of –5.1 eV for vacuum, –4.7 eV for mesitylene and –4.5 eV for water environment.

6.3. Transporting orbitals

(a)

(b)

(c)

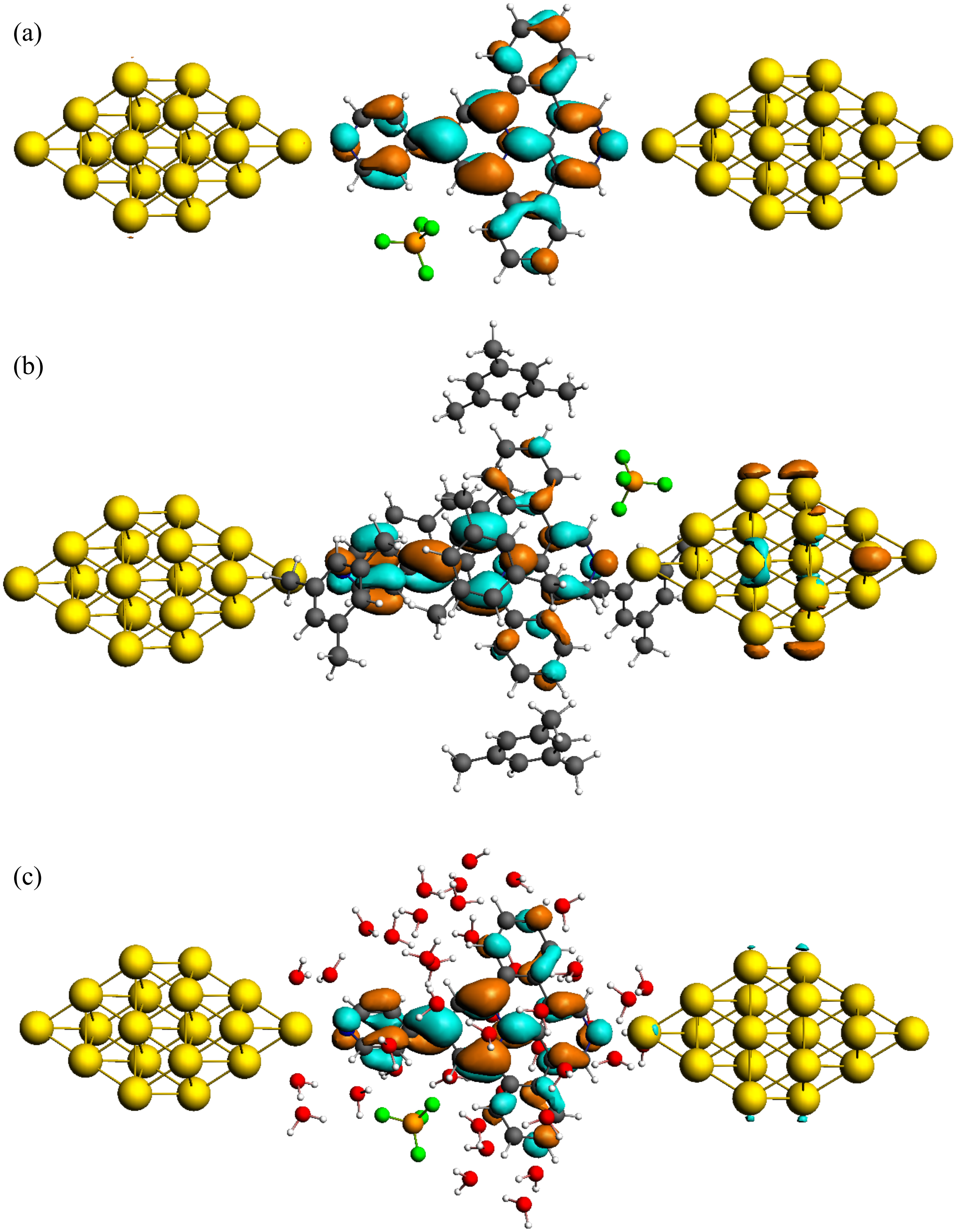

Figure S7. Molecule-localized LUMO (isocontours 0.02) representing the most probable charge transport channel in single molecule junction of **1** in vacuum (a) and with explicit inclusion of 6 mesitylene (b) and 42 water (c) molecules.

7. Single molecule junction of 2 in vacuum, water and mesitylene solvent.

7.1. Optimized molecular junction geometries.

(a)

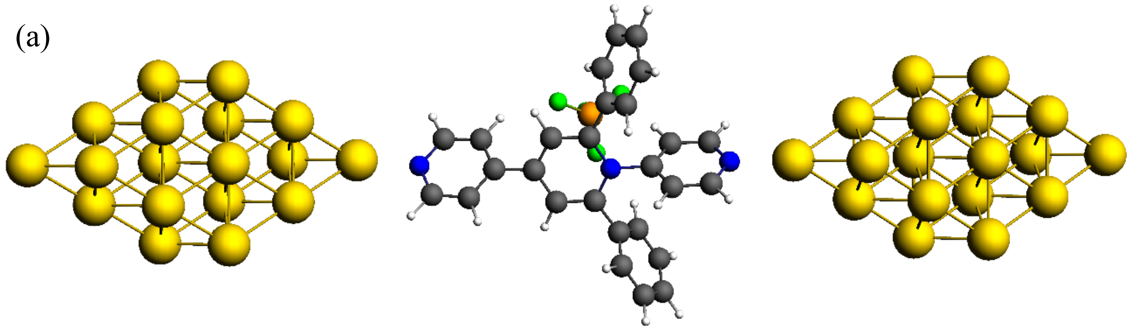

(b)

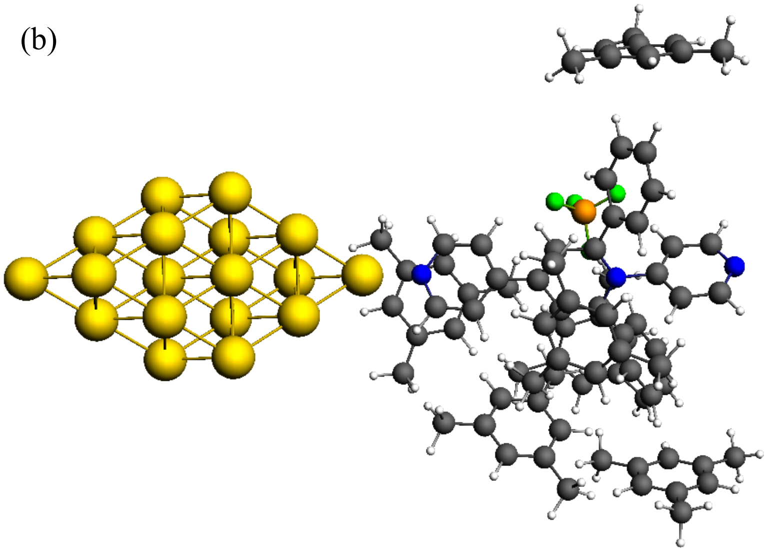

(c)

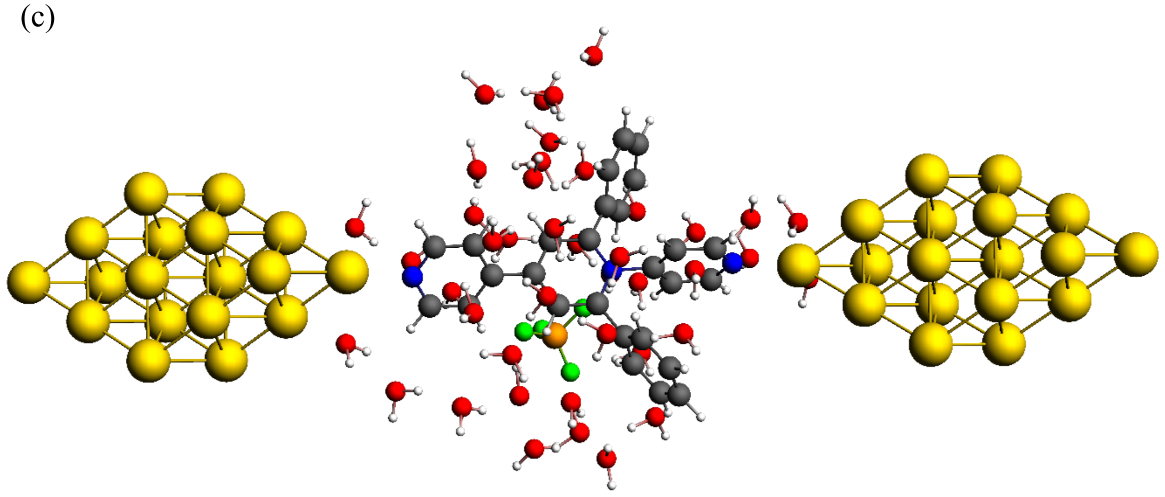

Figure S8. Molecular junction configuration for molecule **2** in vacuum (a) and with explicit inclusion of 6 mesitylene (b) and 42 water (c) molecules.

7.2. Transmission functions

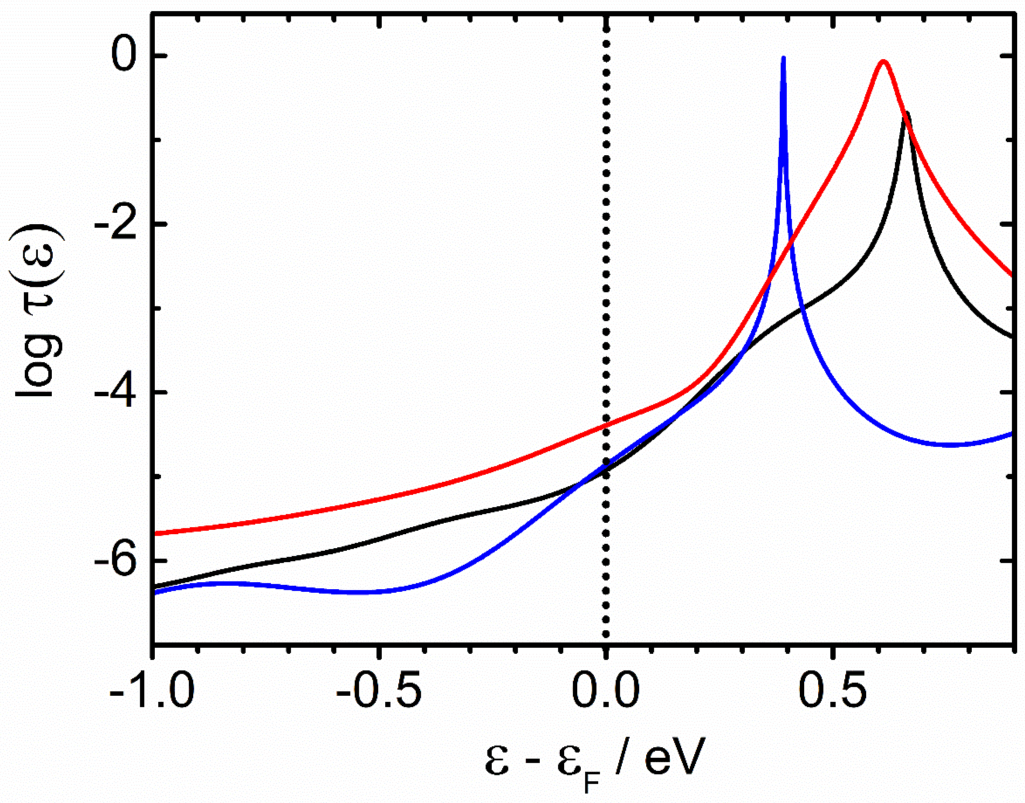

Figure S9. Transmission function for single molecule junction of **2** calculated for vacuum (black), mesitylene (blue) and water (red) environment. Energy axis was scaled using Fermi energy ε_F value of –5.1 eV for vacuum, –4.7 eV for mesitylene and –4.5 eV for water environment.

7.3. Transporting orbitals

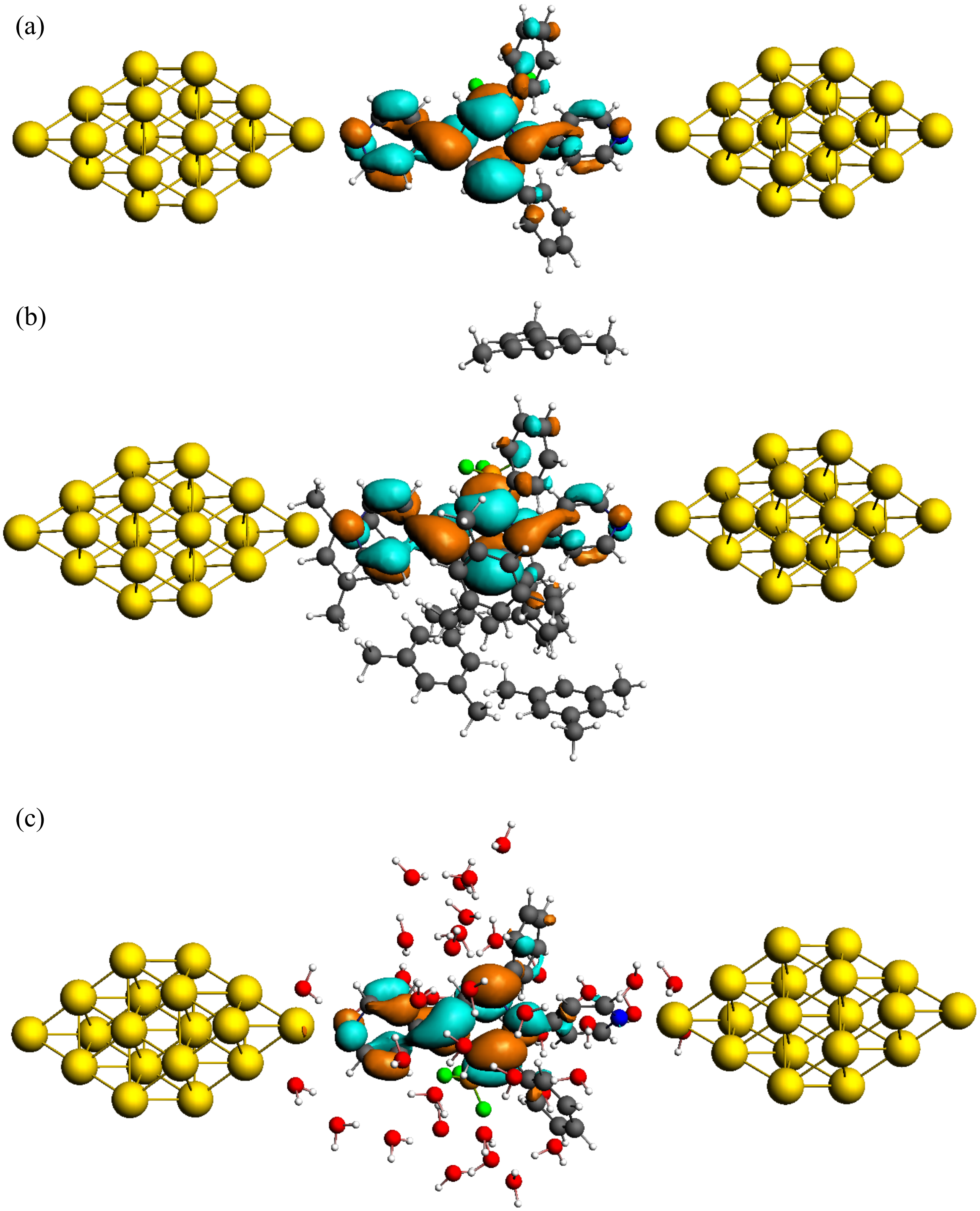

Figure S10. Molecule-localized LUMO (isocontours 0.02) representing the most probable charge transport channel in single molecule junction of **2** in vacuum (a) and with explicit inclusion of 6 mesitylene (b) and 42 water (c) molecules.

8. Single molecule junction of 3 in vacuum, water and mesitylene solvent.

8.1. Optimized molecular junction geometries.

(a)

(b)

(c)

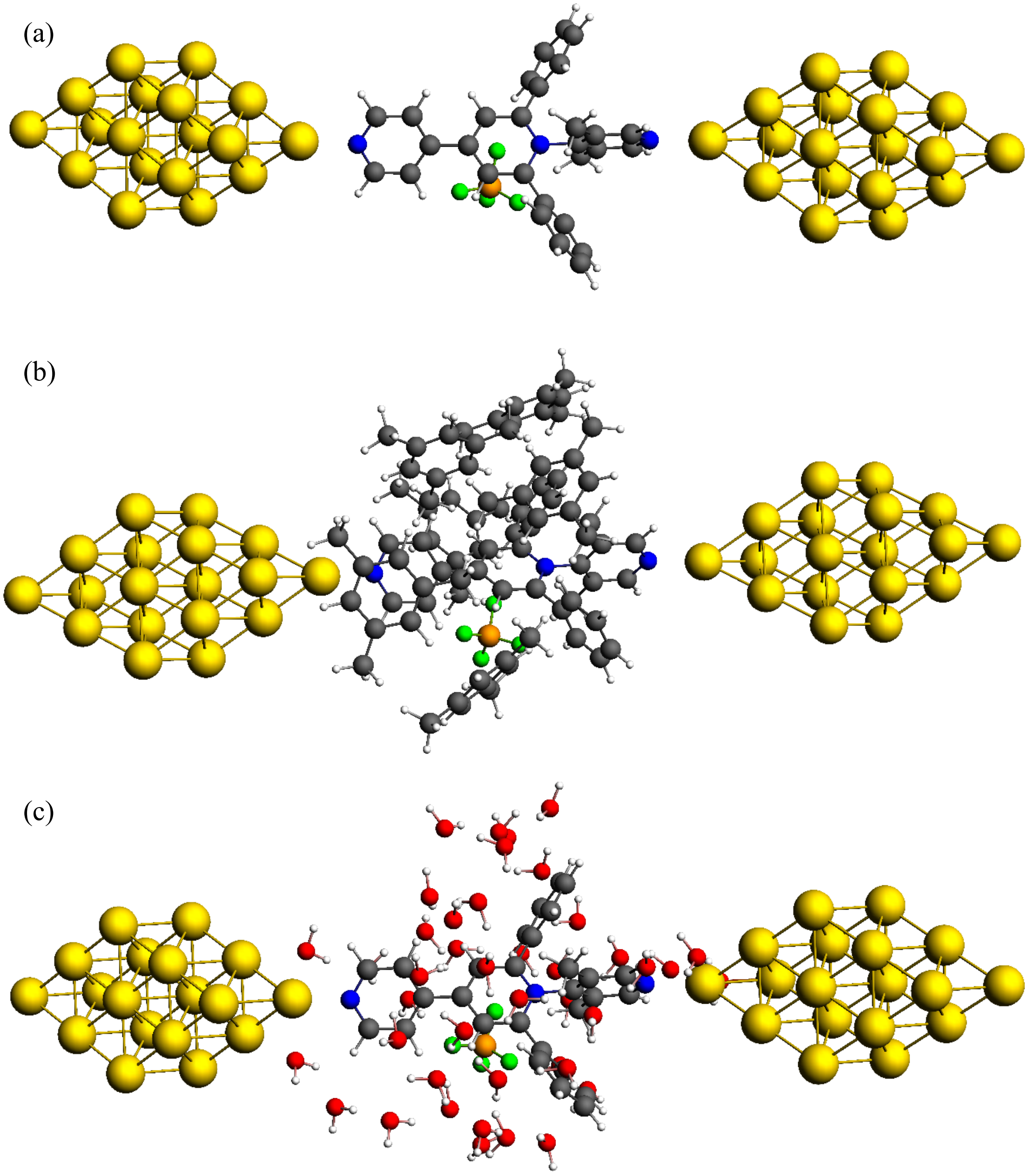

Figure S11. Molecular junction configuration for molecule **3** in vacuum (a) and with explicit inclusion of 6 mesitylene (b) and 42 water (c) molecules.

8.2. Transmission functions

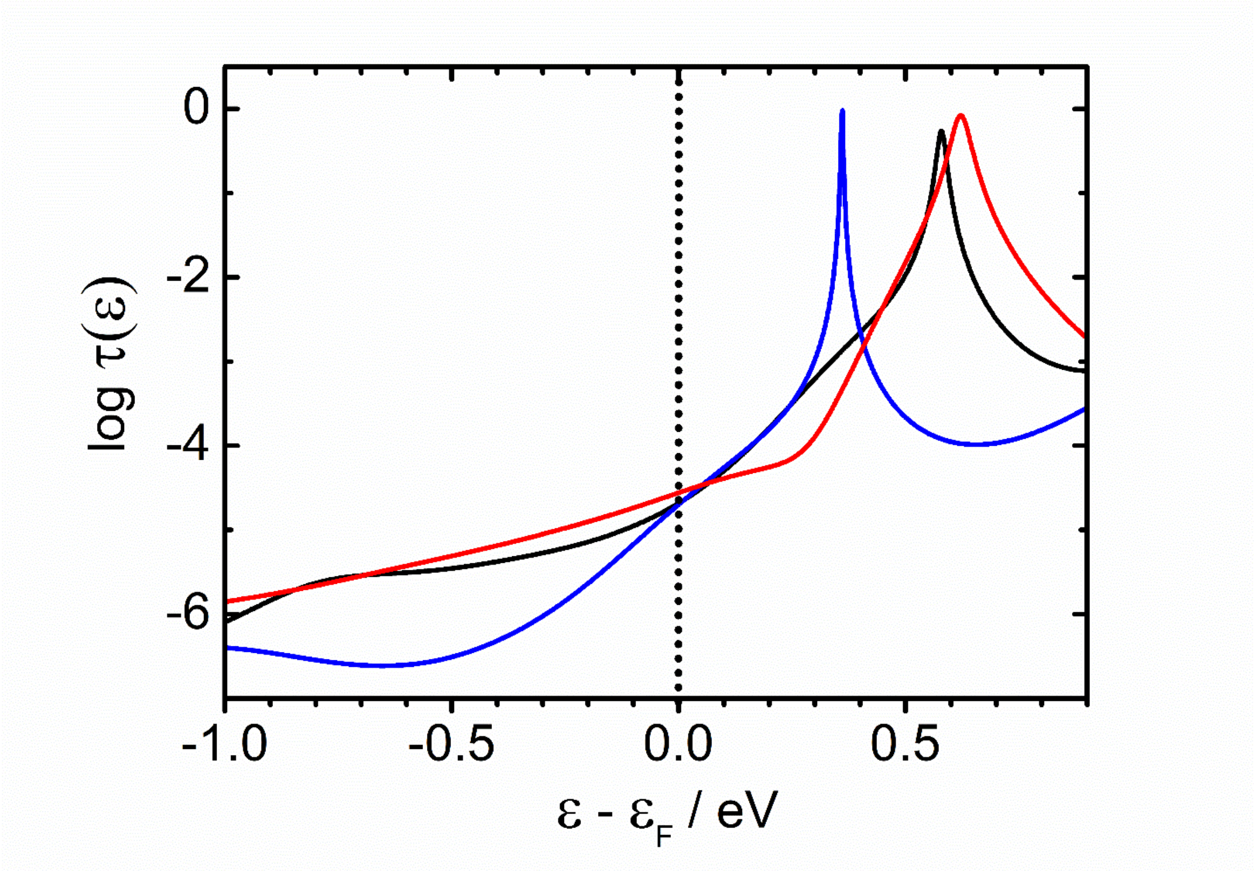

Figure S12. Transmission function for single molecule junction of **3** calculated for vacuum (black), mesitylene (blue) and water (red) environment. Energy axis was scaled using Fermi energy ε_F value of –5.1 eV for vacuum, –4.7 eV for mesitylene and –4.5 eV for water environment.

8.3. Transporting orbitals

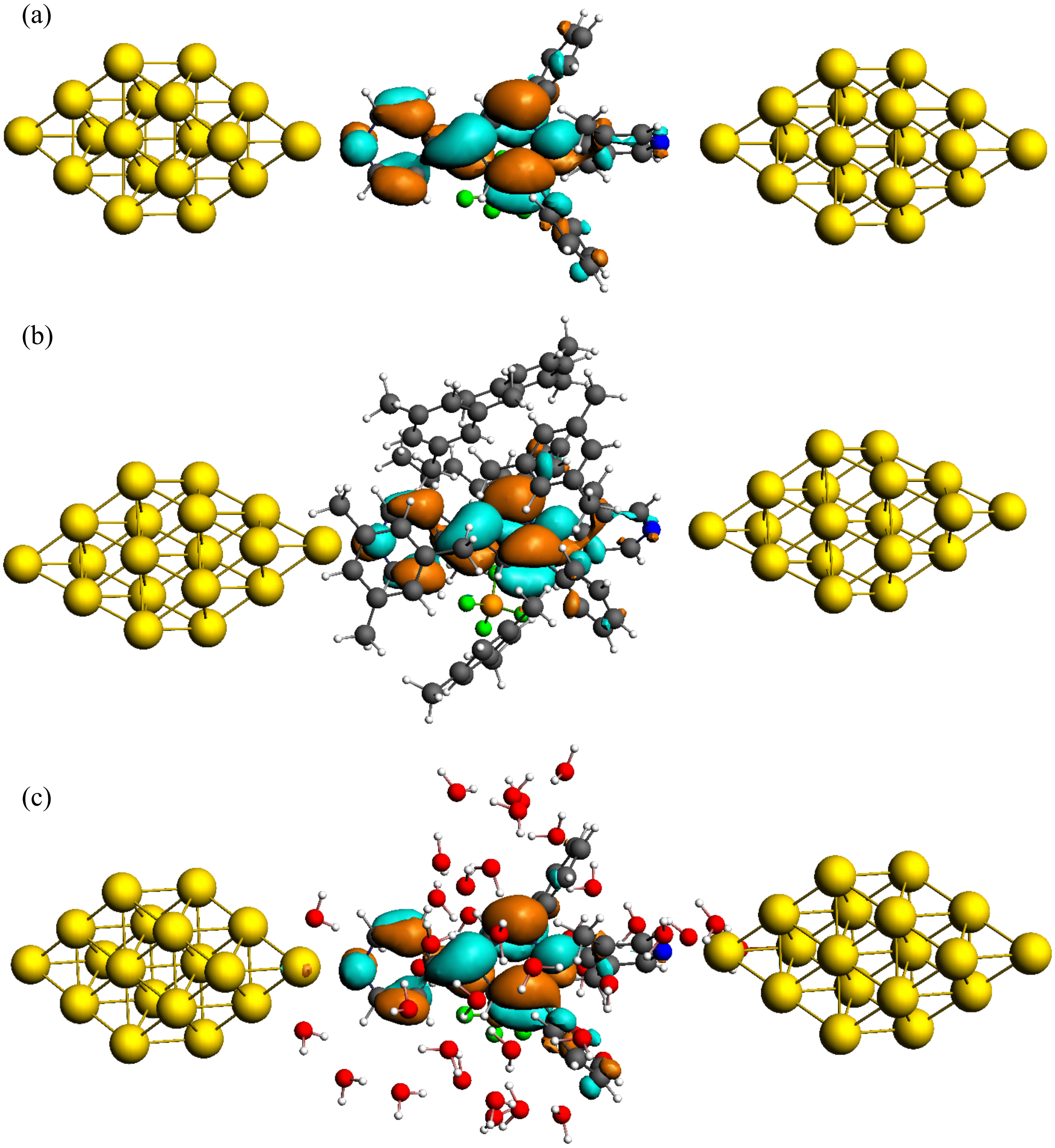

Figure S13. Molecule-localized LUMO (isocontours 0.02) representing the most probable charge transport channel in single molecule junction of **3** in vacuum (a) and with explicit inclusion of 6 mesitylene (b) and 42 water (c) molecules.

9. Single molecule junction of 4 in vacuum, water and mesitylene solvent.

9.1. Optimized molecular junction geometries.

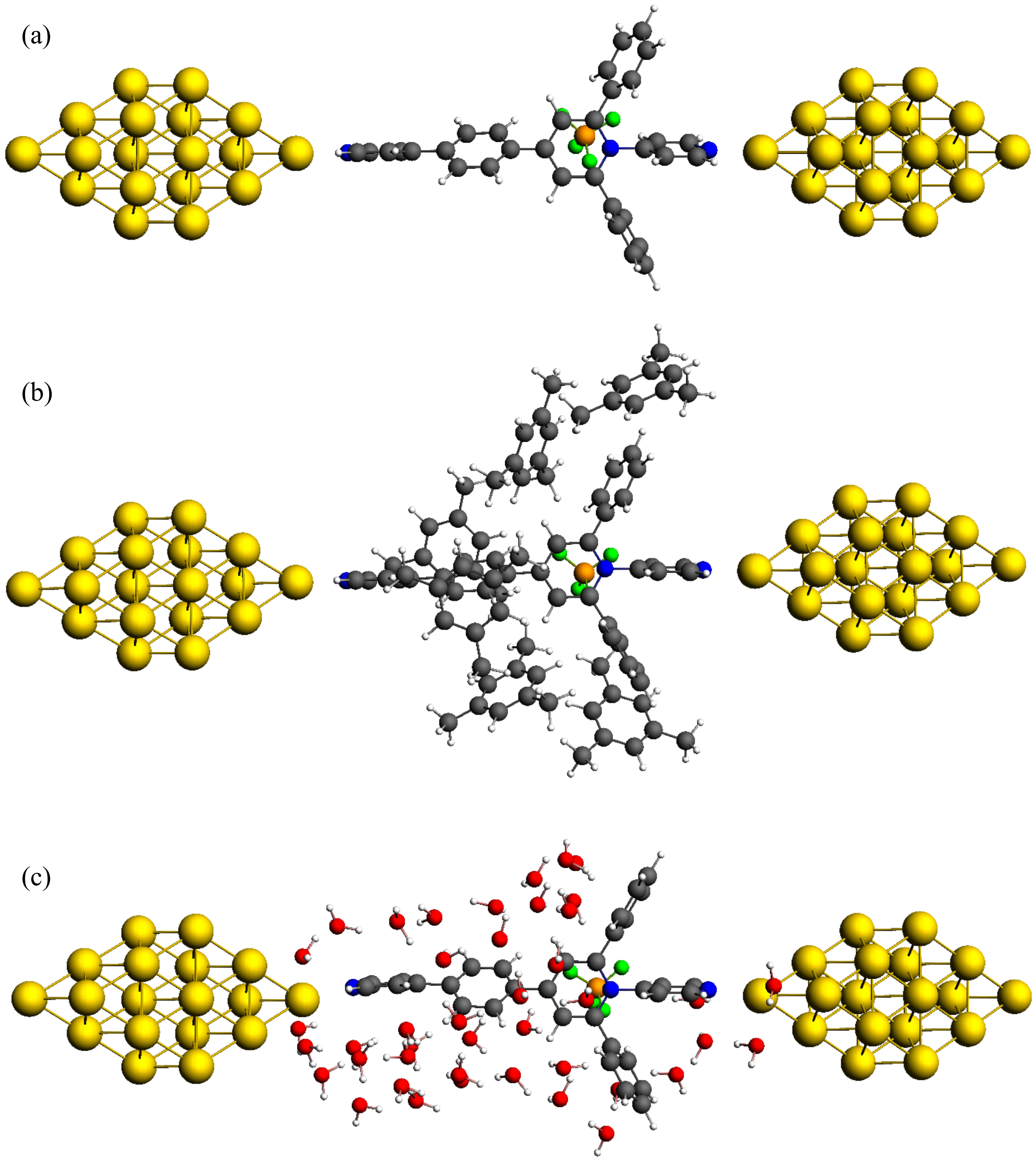

Figure S14. Molecular junction configuration for molecule **4** in vacuum (a) and with explicit inclusion of 6 mesitylene (b) and 42 water (c) molecules.

9.2. Transmission functions

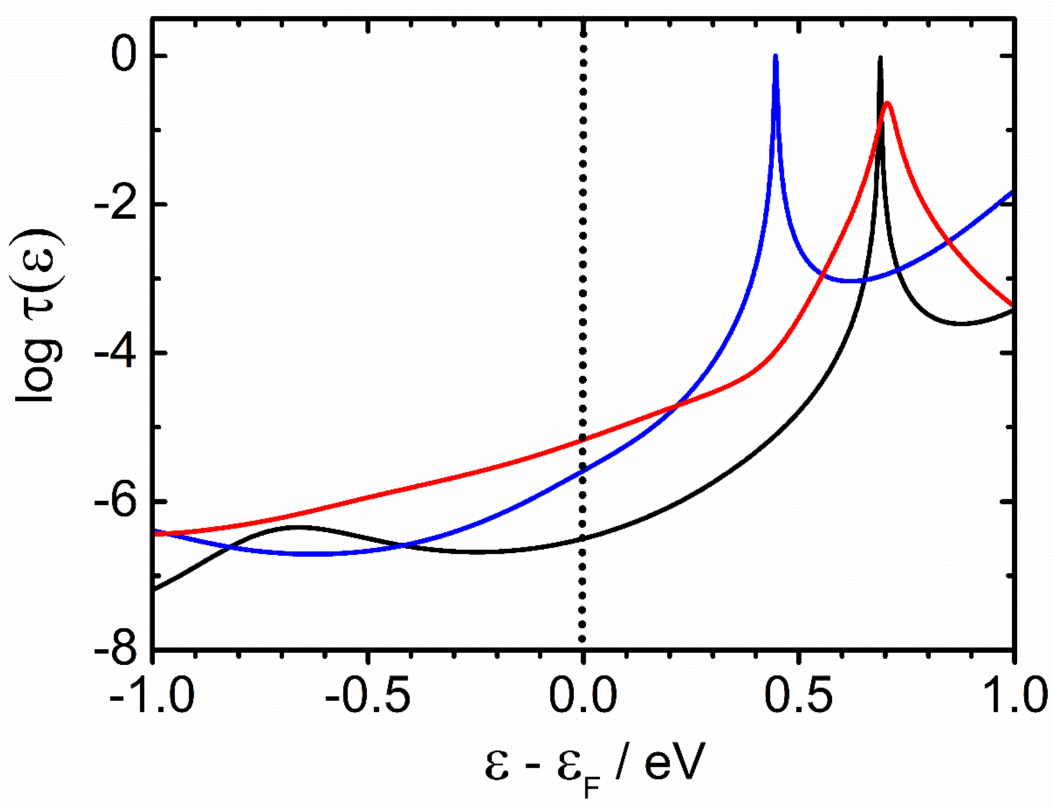

Figure S15. Transmission function for single molecule junction of **4** calculated for vacuum (black), mesitylene (blue) and water (red) environment. Energy axis was scaled using Fermi energy ε_F value of –5.1 eV for vacuum, –4.7 eV for mesitylene and –4.5 eV for water environment.

9.3. Transporting orbitals

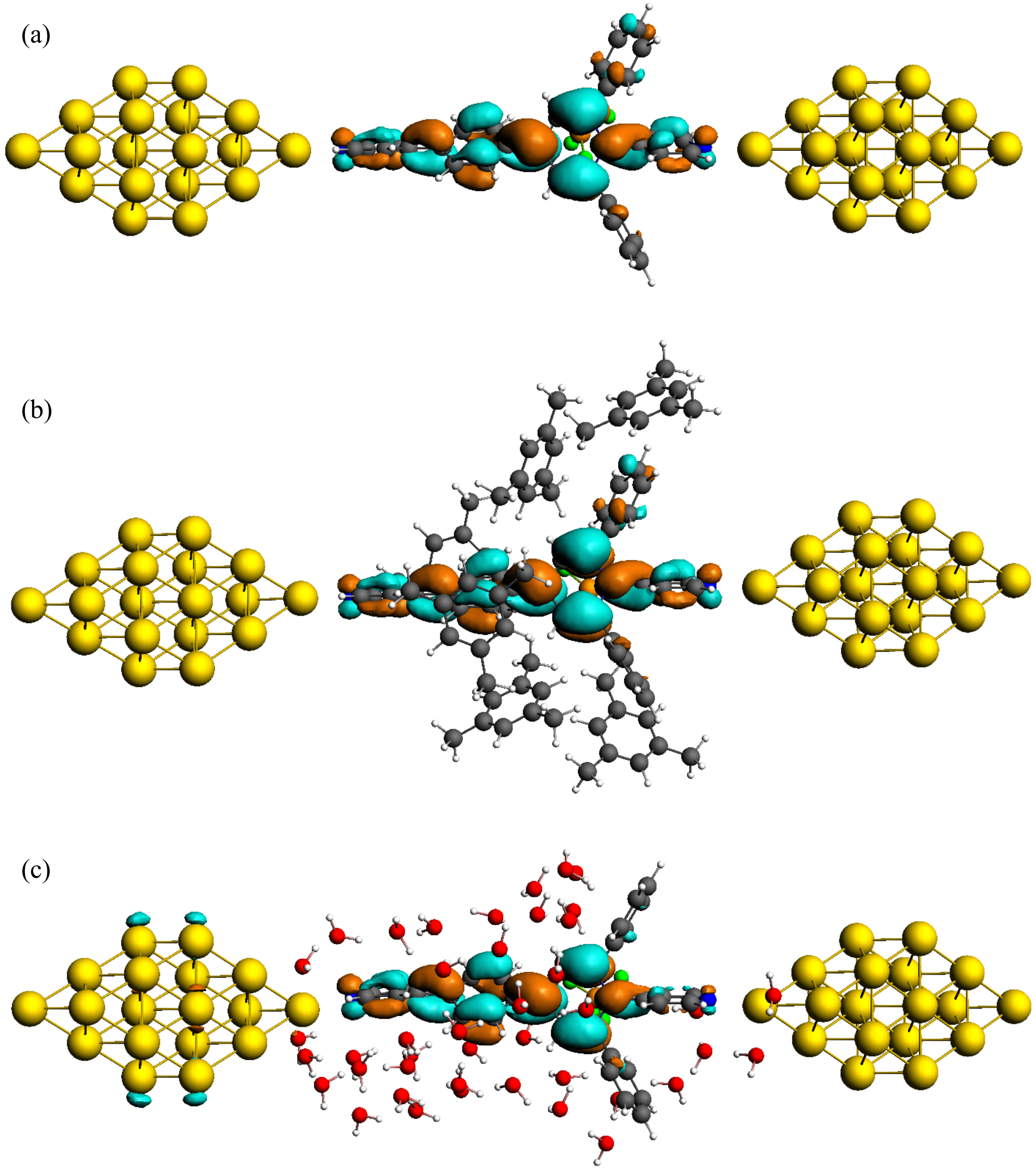

Figure S16. Molecule-localized LUMO (isocontours 0.02) representing the most probable charge transport channel in single molecule junction of **4** in vacuum (a) and with explicit inclusion of 6 mesitylene (b) and 42 water (c) molecules.

10. Transporting orbital energies for optimized geometries.

Table S1. Energies of *molecule-localized* LUMO representing the most probable charge transport channel in fully extended single molecule junctions of **1** to **4** in vacuum, mesitylene and water solvent, ε_F represents the gold Fermi energy in a given solvent.

MJ	ε_{LUMO} /eV	ε_{LUMO}-ε_F /eV	ε_{LUMO} /eV	ε_{LUMO}-ε_F /eV	ε_{LUMO} /eV	ε_{LUMO}-ε_F /eV	z_{th}/nm
	vacuum		mesitylene		water		
**1**	–4.647	+0.453	–4.382	+0.318	–3.928	+0.572	1.32
**2**	–4.516	+0.584	–4.340	+0.360	–3.854	+0.646	1.32
**3**	–4.434	+0.666	–4.309	+0.391	–3.861	+0.639	1.33
**4**	–4.412	+0.688	–4.255	+0.445	–3.763	+0.737	1.74

The *molecule-localized* LUMO is equal to LUMO of the extended molecular junction system for all studied molecules **1** to **4** and thus has the same energy. Details on the computational method and quantum mechanical methodology are given in Section 5.

11. Theoretical single molecule conductances.

Table S2. Summary of the calculated conductance and MJ length values for all investigated molecular junctions.

MJ	log $(G/G_0)^{th}$			z_{th} /nm	log $(G/G_0)^{th}$	z_{th} /nm[d]
	vacuum[a]	water[b]	mesitylene[c]		mesitylene[c]	
**1**	–2.89	–3.57	–2.70	1.32	–3.61	1.10
**2**	–4.68	–4.56	–4.70	1.32	–4.55	1.00
**3**	–4.92	–4.39	–4.86	1.33	–4.13[e]	1.00[e]
**4**	–6.50	–5.17	–5.59	1.74	–4.87	1.20

[a] calculated for ε_F = –5.1 eV
[b] calculated for ε_F = –4.5 eV
[c] calculated for ε_F = –4.7 eV
[d] MJ length was fixed to z_{exp} value
[e] initial configuration was the same as for **2**

Table S3. Effect of the choice of Fermi energy ε_F value on the calculated conductance for fully extended MJ geometries.

MJ	log $(G/G_0)^{exp}$	log $(G/G_0)^{th}$						
		–4.90[a]	–4.85[a]	–4.80[a]	–4.75[a]	–4.70[a,b]	–4.65[a]	–4.60[a]
**1**	–4.14 ± 0.48	–3.66	–3.49	–3.28	–3.02	–2.70	–2.32	–1.90
**2**	–4.72 ± 0.51	–5.63	–5.41	–5.17	–4.93	–4.70	–4.47	–4.26
**3**	–4.72 ± 0.52	–5.68	–5.47	–5.27	–5.06	–4.86	–4.67	–4.49
**4**	–5.23 ± 0.44	–6.18	–6.05	–5.90	–5.75	–5.59	–5.42	–5.25

[a] ε_F value used
[b] selected ε_F value based on the smallest sum $\sum_1^4 \left|\log(G/G_0)^{exp} - \log(G/G_0)^{theor}\right|$

Table S4. Effect of the choice of Fermi energy ε_F value on the calculated conductance for MJ length fixed to the experimental value z_{exp}.

MJ	log $(G/G_0)^{exp}$	log $(G/G_0)^{th}$						
		–4.90[a]	–4.85[a]	–4.80[a,b]	–4.75[a]	–4.70[a]	–4.65[a]	–4.60[a]
**1**	–4.14 ± 0.48	–4.32	–4.17	–4.00	–3.81	–3.61	–3.39	–3.18
**2**	–4.72 ± 0.51	–5.00	–4.92	–4.82	–4.70	–4.55	–4.36	–4.12
**3**[c]	–4.72 ± 0.52	–4.99	–4.80	–4.60	–4.37	–4.13	–3.87	–3.56
**4**	–5.23 ± 0.44	–5.62	–5.42	–5.23	–5.04	–4.87	–4.75	–4.68

[a] ε_F value used
[b] selected ε_F value based on the smallest sum $\sum_1^4 \left|\log(G/G_0)^{exp} - \log(G/G_0)^{theor}\right|$
[c] z_{th} = 1.10 nm, initial configuration was the same as for **2**

12. Torsion angle effect

Geometry optimized molecular junction geometries including explicit water molecules describe experimental single molecule conductance for all studied molecules quite accurately. Single molecule conductance values of molecular junctions after explicit inclusion of mesitylene solvent agrees with experimental values for molecules **2** to **4**, but highly overestimates the experimental value for molecule **1** in mesitylene ($\log(G/G_0)^{th}$ = –2.70 versus $\log(G/G_0)^{exp}$ = –4.14 ± 0.48). In order to explain this discrepancy, we investigated the influence of the rotation between the planar pyridinium center and adjacent pyridine anchoring group in molecule **1** on the computed conductance as defined in Figure S17. For the geometry optimized molecular junction shown in Figure S5b the torsion angle θ_4 is 34.7°. Table S5 summarizes $\log(G/G_0)^{th}$ values for selected geometries with fixed torsion angle θ_4.

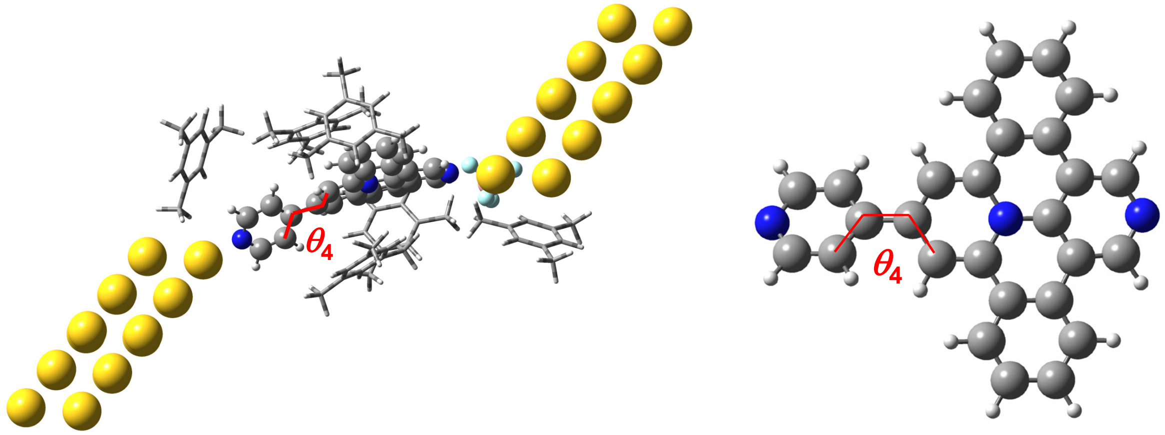

Figure S17. The definition of torsion angle θ_4 (red) for molecular junction of **1**.

Table S5. Summary of calculated conductance $\log(G/G_0)$ for molecular junction of **1** in mesitylene at different torsional angles θ_4

MJ	θ_4 /°	log $(G/G_0)^{th}$	z_{th} /nm
**1**	0.0	–2.10[a]	1.32
**1**	34.7[b]	–2.70	1.32
**1**	45.0	–2.94	1.32
**1**	60.0	–3.38	1.32
**1**	75.0	–3.96	1.32
**1**	90.0	–4.99	1.32

[a] all conductance values in mesitylene are calculated for ε_F = –4.7 eV
[b] value for geometry optimized structure without constrained angle θ_4

13. Molecular junction geometries in mesitylene at experimental junction length

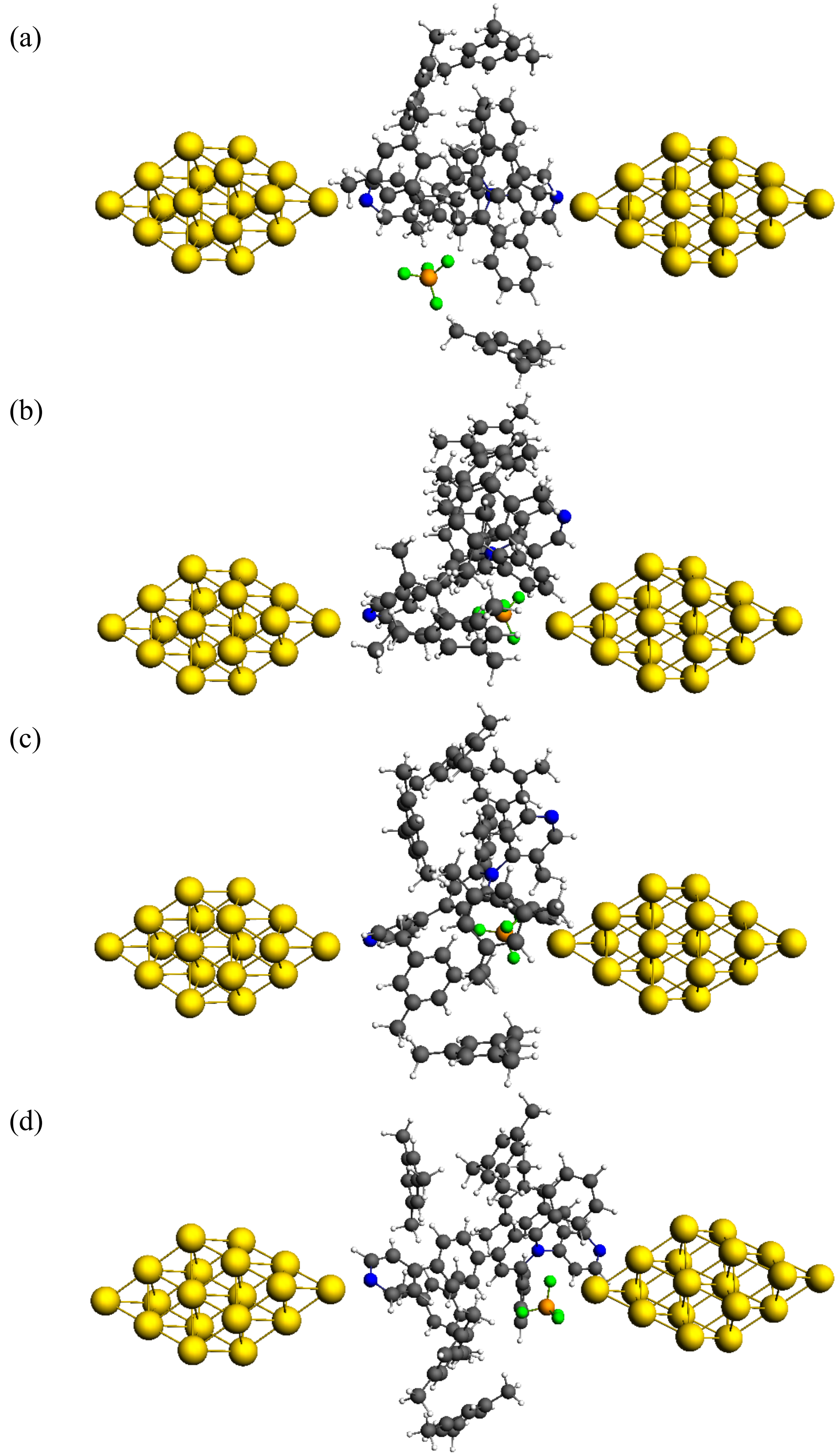

Figure S18. Molecular junction geometries for molecules **1** (a), **2** (b), **3** (c) and **4** (d) in mesitylene calculated for experimentally determined distance between gold electrodes.

14. Stabilization and interaction energies for adsorbed molecules

DFT stabilization energies were calculated based on geometry optimized structures depicted in Figure S19 and their values are summarized in Table S6. Calculations indicate that cation **1**$^+$ due to its planar structure can be more energetically stabilized on the Au(111) surface compared to cations **2**$^+$ and **3**$^+$.

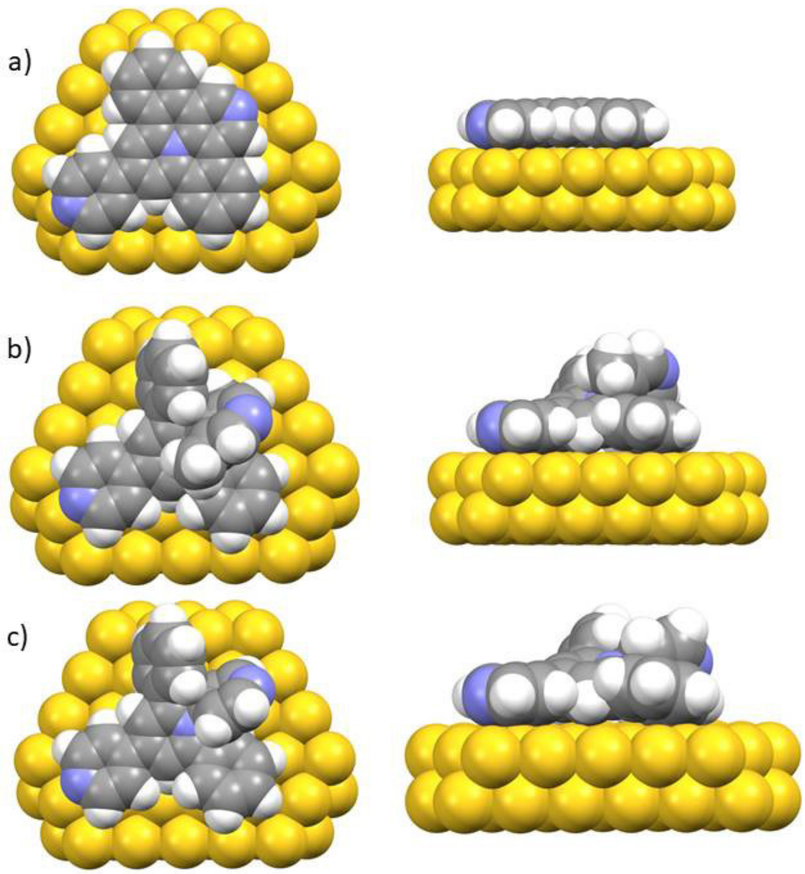

Figure S19. The geometry optimized structures of a) **1**$^+$-electrode, b) **2**$^+$-electrode and c) **3**$^+$-electrode using PBE-D3/def-SVP/def-ecp level in vacuum.

Table S6. DFT calculated stabilization energies ΔE (kcal/mol) and interaction energies ΔE_{int} (kJ/mol) using B3LYP-D3/6-311G(d,p)/LANL2DZ level in vacuum.

System	ΔE (kcal/mol)	ΔE_{int} (kJ/mol)
**1**$^+$-electrode	91.0	–380.7
**2**$^+$-electrode	71.5	–299.3
**3**$^+$-electrode	68.1	–285.1

15. References.

(1) Nováková Lachmanová, Š.; Šebera, J.; Kolivoška, V.; J. Gasior, G.; Mészáros, G.; Dupeyre, P.; Lainé, P.; Hromadová, M. Correlation of Electrochemical Properties of Expanded Pyridinium Compounds with their Single Molecule Conductance. *Electrochim. Acta* **2018**, *264*, 301–311.

(2) Mészáros, G.; Li, Ch.; Pobelov, I.; Wandlowski, T. Current Measurements in a Wide Dynamic Range-Applications in Electrochemical Nanotechnology. *Nanotechnology* **2007**, *18*, 424004.

(3) Hong, W.; Valkenier, H.; Meszaros, G.; Manrique, D. Z.; Mishchenko, A.; Putz, A.; Moreno-García, P.; Lambert, C. J.; Hummelen, J. C.; Wandlowski, T. An MCBJ Case Study: The Influence of π-Conjugation on the Single-Molecule Conductance at a Solid/Liquid Interface. *Beilstein J. Nanotechnol.* **2011**, *2*, 699−713.

(4) Šebera, J.; Kolivoška, V.; Valášek, M.; Gasior, J; Sokolová, R.; Mészáros, G.; Hong, W.; Mayor, M.; Hromadová, M. Tuning Charge Transport Properties of Asymmetric Molecular Junctions. *J. Phys. Chem. C* **2017**, *121*, 12885−12894.

(5) Zhao, J.; Murakoshi, K.; Yin, X.; Kiguchi, M.; Guo, Y.; Wang, N.; Liang, S.; Liu, H. Dynamic Characterization of the Postbreaking Behavior of a Nanowire. *J. Phys. Chem. C* **2008,** *112,* 20088–20094.

(6) Becke, A. D. Density-Functional Thermochemistry. 3. The Role of Exact Exchange. *J. Chem. Phys.* **1993**, *98*, 5648–5652.

(7) Lee, C. T.; Yang, W. T.; Parr, R. G. Development of the Colle-Salvetti Correlation-Energy Formula into a Functional of the Electron-Density. *Phys. Rev. B* **1988**, *37*, 785−789.

(8) Van Tendeloo, G.; Van Dyck, D.; Pennycook, S. J., *Handbook of Nanoscopy*. John Wiley & Sons: 2012.

(9) Harihara, P. C.; Pople, J. A. Influence of Polarization Functions on Molecular-Orbital Hydrogenation Energies. *Theor. Chim. Acta* **1973**, *28*, 213−222.

(10) Hay, P. J.; Wadt, W. R. Ab-initio Effective Core Potentials for Molecular Calculations - Potentials for K to Au Including the Outermost Core Orbitals. *J. Chem. Phys.* **1985**, *82*, 299−310.

(11) Milan, D. C.; Al-Owaedi, O. A.; Oerthel, M. C.; Marques-Gonzalez, S.; Brooke, R. J.; Bryce, M. R.; Cea, P.; Ferrer, J.; Higgins, S. J.; Lambert, C. J.; Low, P. J.; Manrique, D. Z.; Martin, S.; Nichols, R. J.; Schwarzacher, W.; Garcia-Suarez, V. M. Solvent Dependence of the Single

Molecule Conductance of Oligoyne-Based Molecular Wires. *J. Phys. Chem. C* **2016**, *120*, 15666−15674.

(12) Dapprich, S.; Komaromi, I.; Byun, K. S.; Morokuma, K.; Frisch, M. J. A new ONIOM implementation in Gaussian98. Part I. The Calculation of Energies, Gradients, Vibrational Frequencies and Electric Field Derivatives. *Theochem-J. Mol. Struct.* **1999**, *461*, 1−21.

(13) Frisch, M. J.; Trucks, G. W.; Schlegel, H. B.; Scuseria, G. E.; Robb, M. A.; Cheeseman, J. R.; Scalmani, G.; Barone, V.; Petersson, G. A.; Nakatsuji, H.; Li, X.; Caricato, M.; Marenich, A. V.; Bloino, J.; Janesko, B. G.; Gomperts, R.; Mennucci, B.; Hratchian, H. P.; Ortiz, J. V.; Izmaylov, A. F.; Sonnenberg, J. L.; Williams; Ding, F.; Lipparini, F.; Egidi, F.; Goings, J.; Peng, B.; Petrone, A.; Henderson, T.; Ranasinghe, D.; Zakrzewski, V. G.; Gao, J.; Rega, N.; Zheng, G.; Liang, W.; Hada, M.; Ehara, M.; Toyota, K.; Fukuda, R.; Hasegawa, J.; Ishida, M.; Nakajima, T.; Honda, Y.; Kitao, O.; Nakai, H.; Vreven, T.; Throssell, K.; Montgomery Jr., J. A.; Peralta, J. E.; Ogliaro, F.; Bearpark, M. J.; Heyd, J. J.; Brothers, E. N.; Kudin, K. N.; Staroverov, V. N.; Keith, T. A.; Kobayashi, R.; Normand, J.; Raghavachari, K.; Rendell, A. P.; Burant, J. C.; Iyengar, S. S.; Tomasi, J.; Cossi, M.; Millam, J. M.; Klene, M.; Adamo, C.; Cammi, R.; Ochterski, J. W.; Martin, R. L.; Morokuma, K.; Farkas, O.; Foresman, J. B.; Fox, D. J. *Gaussian 09 Rev. D.01*, Wallingford, CT, 2009.

(14) Verzijl, C. J. O.; Seldenthuis, J. S.; Thijssen, J. M. Applicability of the Wide-Band Limit in DFT-Based Molecular Transport Calculations. *J. Chem. Phys.* **2013**, *138*, 094102.

(15) Verzijl, C. J. O.; Thijssen, J. M. DFT-Based Molecular Transport Implementation in ADF/BAND. *J. Phys. Chem. C* **2012**, *116*, 24393−24412.

(16) te Velde, G.; Bickelhaupt, F. M.; Baerends, E. J.; Guerra, C. F.; Van Gisbergen, S. J. A.; Snijders, J. G.; Ziegler, T. Chemistry with ADF. *J. Comput. Chem.* **2001**, *22*, 931−967.

(17) Reiher, M.; Salomon, O.; Hess, B. A., Reparameterization of Hybrid Functionals Based on Energy Differences of States of Different Multiplicity. *Theor. Chem. Acc.* **2001**, *107*, 48−55.

(18) van Lenthe, E.; Baerends, E. J. Optimized Slater-Type Basis Sets for the Elements 1-118. *J. Comput. Chem.* **2003**, *24*, 1142−1156.

(19) van Lenthe, E.; van Leeuwen, R.; Baerends, E. J.; Snijders, J. G., Relativistic Regular Two-Component Hamiltonians. *Int. J. Quantum Chem.* **1996**, *57*, 281−293

(20) Landauer, R. Conductance Determined by Transmission–Probes and Quantized Constriction Resistance. *J. Phys. Condens. Matter* **1989**, *1*, 8099−8110.

(21) Datta, S. *Electronic Transport in Mesoscopic Systems*. Cambridge university press: 1997.

(22) Veenstra, S. C.; Stalmach, U.; Krasnikov, V. V.; Hadziioannou, G.; Jonkman, H. T.; Heeres, A.; Sawatzky, G. A. Energy Level Alignment at the Conjugated Phenylenevinylene Oligomer/Metal Interface. *Appl. Phys. Lett.* **2000**, *76*, 2253−2255.

(23) Bâldea, I. Transition Voltage Spectroscopy Reveals Significant Solvent Effects on Molecular Transport and Settles an Important Issue in Bipyridine-Based Junctions. *Nanoscale* **2013**, *5*, 9222−9230.

(24) Heras, J. M.; Viscido, L. Work Function Changes upon Water Contamination of Metal Surfaces. *Appl. Surf. Sci.* **1980**, *4*, 238–241.

(25) Frisenda, R.; Janssen, V. A. E. C.; Grozema, F. C.; van der Zant, H. S. J.; Renaud. N. Mechanically Controlled Quantum Interference in Individual π-Stacked Dimers. *Nature Chem.* **2016**, *8*, 1099−1104.

(26) Perdew, J. P.; Wang, Y. Accurate and Simple Analytic Representation of the Electron-Gas Correlation-Energy. *Phys. Rev. B* **1992**, *45*, 13244−13249.

(27) Perdew, J. P.; Burke, K.; Ernzerhof, M. Generalized Gradient Approximation Made Simple. *Phys. Rev. Lett.* **1996**, *77*, 3865−3868.

(28) Grimme, S.; Antony, J.; Ehrlich, S.; Krieg, H. A Consistent and Accurate Ab Initio Parametrization of Density Functional Dispersion Correction (DFT-D) for the 94 elements H-Pu. *J. Chem. Phys.* **2010**, *132*, 154104.

(29) Eichkorn, K.; Treutler, O.; Ohm, H.; Haser, M.; Ahlrichs, R. Auxiliary Basis-Sets to Approximate Coulomb Potentials. *Chem. Phys. Lett.* **1995**, *240*, 283−289.

(30) Ahlrichs, R.; Bar, M.; Haser, M.; Horn, H.; Kolmel, C. Electronic-Structure Calculations on Workstation Computers - the Program System Turbomole. *Chem. Phys. Lett.* **1989**, *162*, 165−169.

(31) Schafer, A.; Horn, H.; Ahlrichs, R. Fully Optimized Contracted Gaussain-Basis Sets for Atoms Li to Kr. *J. Chem. Phys.* **1992**, *97*, 2571−2577.

(32) Andrae, D.; Haussermann, U.; Dolg, M.; Stoll, H.; Preuss, H. Energy-Adjusted Ab initio Pseudopotentials for the 2nd and 3rd Row Transition-Elemenets. *Theor. Chim. Acta* **1990**, *77*, 123−141.

(33) Krishnan, R.; Binkley, J. S.; Seeger, R.; Pople, J. A. Self-Consistent Molecular-Orbital Methods. 20. Basis Set for Correlated Wave-Functions. *J. Chem. Phys.* **1980**, *72*, 650−654.
